# The efficacy and safety of oral antibiotic treatment in patients with chronic low back pain and Modic changes: A systematic review and meta‐analysis

**DOI:** 10.1002/jsp2.1281

**Published:** 2023-09-19

**Authors:** Arnold Y. L. Wong, G. Michael Mallow, Sabina M. Pinto, Alexander L. Hornung, Samuel S. Rudisill, Khaled Aboushaala, Peter M. Udby, Howard S. An, Dino Samartzis

**Affiliations:** ^1^ Department of Rehabilitation Sciences The Hong Kong Polytechnic University Hung Hom, Hong Kong SAR China; ^2^ Department of Orthopedic Surgery Rush University Medical Center Chicago Illinois USA; ^3^ Spine Unit Rigshospitalet, University of Copenhagen Copenhagen Denmark

**Keywords:** low back, meta‐analysis, Modic change, nucleus pulposus, pain, pain antibiotics, systematic review

## Abstract

**Background:**

This systematic review and meta‐analysis aimed to summarize evidence regarding the effectiveness and safety of oral antibiotic intervention for chronic low back pain (CLBP) patients with/without type‐1 Modic changes (MC1).

**Methods:**

AMED, CINAHL, Cochrane Library, Embase, and Medline were searched from inception to March 3, 2023. Randomized controlled trials (RCTs) or non‐RCTs that investigated the effectiveness or safety of oral antibiotics in treating CLBP patients were eligible for inclusion. Two independent reviewers screened abstracts, full‐text articles, and extracted data. The methodological quality of each included article were evaluated by RoB2 and NIH quality assessment tools. The quality of evidence was appraised by GRADE. Meta‐analyses were performed, where applicable. A subgroup analysis was conducted to evaluate the RCTs and case series separately, and to evaluate the effect of removing a low‐quality RCT.

**Results:**

Three RCTs and four case series were included. All Amoxicillin‐clavulanate/Amoxicillin treatments lasted for approximately 3 months. Moderate‐ and low‐quality evidence suggested that antibiotic was significantly better than placebo in improving disability and quality of life in CLBP patients with MC1 at 12‐month follow‐up, respectively. Low‐quality evidence from meta‐analyses of RCTs showed that oral antibiotic was significantly better than placebo in improving pain and disability in CLBP patients with MC1 immediately post‐treatment. Very low‐quality evidence from the case series suggested that oral Amoxicillin‐clavulanate significantly improved LBP/leg pain, and LBP‐related disability. Conversely, low‐quality evidence found that oral Amoxicillin alone was not significantly better than placebo in improving global perceived health in patients with CLBP at the 12‐month follow‐up. Additionally, oral antibiotic users had significantly more adverse effects than placebo users.

**Conclusions:**

Although oral antibiotics were statistically superior to placebo in reducing LBP‐related disability in patients with CLBP and concomitant MC1, its clinical significance remains uncertain. Future large‐scale high‐quality RCTs are warranted to validate the effectiveness of antibiotics in individuals with CLBP.

## Graphical abstract

This systematic review and meta‐analysis aimed to summarize evidence regarding the effectiveness and safety of oral antibiotic intervention for chronic low back pain (CLBP) patients with/without type‐1 Modic changes (MC1). Although oral antibiotics were statistically superior to placebo in reducing LBP‐related disability in patients with CLBP and concomitant MC1, its clinical significance remains uncertain. Future large‐scale high‐quality RCTs are warranted to validate the effectiveness of antibiotics in individuals with CLBP.

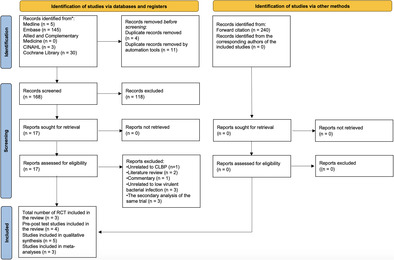

## 1 INTRODUCTION

Low back pain (LBP) is the most disabling condition worldwide.^1^ Many efforts have been made to manage this growing global healthcare problem and associated socioeconomic consequences.^2^ Notably, chronic LBP (CLBP) and the treatment thereof have demonstrated a substantial challenge to modern healthcare providers.^3^ Pharmacological, conservative, and surgical interventions for LBP have their own disadvantages, although their clinical benefits are not fully satisfactory.^3^ For instance, the common use of opioid for treating patients with CLBP may lead to the opioid pandemic, which highlights the importance of better understanding the condition and developing alternative management strategies.^4^

Multiple factors can lead to LBP.^5^, ^6^, ^7^ Knowledge of specific pain generators is needed to improve individualized treatments for patients with LBP. Of various spine phenotypes, Modic changes (MCs) could be a potential target for more personalized treatment for patients with CLBP. MCs are vertebral bone marrow changes visualized by magnetic resonance imaging (MRI).^8^ MCs were first described by de Roos et al.^9^ on MRI in 1987. Modic et al.^8^ then classified MCs into three categories based on T1‐ and T2‐weighted MRI and histological analysis. MC type 1 (MC1) represents reactive/inflammatory changes in the marrow; MC2 consists of yellow lipid marrow replacement; and MC3 is a feature of calcification within the endplate and subchondral vertebral marrow.^8^ MC1 and to a lesser extent MC2 has been associated with both LBP and disability.^10^, ^11^

MCs are common among patients with LBP. The prevalence of MCs in patients with lumbar disc herniation and CLBP was up to 45%, while that in the general adult population was 5%.^12^, ^13^ Population‐ and patient‐based studies have demonstrated a close association between MCs and LBP.^11^, ^14^ Recently, the International Classification of Diseases 10th revision (ICD‐10) and the ICD‐11 have included a specific diagnosis for patients with LBP and concomitant endplate/MCs phenotype—vertebrogenic LBP.^15^ This underscores the importance of the endplate and marrow changes in LBP with vertebrogenic origins. Accordingly, advances in the treatment of LBP patients with MCs could have major impacts on back pain and the associated global health burden.^16^

The etiology of MCs is linked with pathophysiological changes in the endplate and discus causing biomechanical changes in the functional spinal unit (FSU).^5^ Multiple factors (e.g., degenerative changes, inflammation, trauma, iatrogenic MCs after discectomy, and bacterial infection) could increase biomechanical strains in the FSU, resulting in the onset of MCs.^5^, ^17^, ^18^, ^19^, ^20^, ^21^, ^22^ Of various potential causes, the hypothesis of bacterial infection, especially by *Cutibaterium acnes* (*C acnes*), has been drawing a lot of attention. *C acnes* has been found in disc tissues and speculated as a culprit caused by hematogenous spread to the discus/vertebra complex.^23^ Such findings and microbiome analysis illustrate the potential cause of LBP by infection.^24^ Unlike bacterial disc infections (e.g., spondylodiscitis), which are characterized by an acute systemic infection,^25^ an ongoing low virulent infection in the discus/vertebral complex without systemic manifestation may cause symptomatic disc degeneration.^26^ It may also partly explain the development of intervertebral edema with upregulation of a localized inflammatory response in the adjacent vertebra.^5^, ^24^, ^27^

Several randomized controlled trials (RCTs) and case series have investigated the effect of oral antibiotics on LBP intensity and LBP‐related disability in patients with concomitant CLBP and MCs.^28^, ^29^, ^30^ Findings from these studies have caused considerable debate regarding management of CLBP.^31^ While oral antibiotics may significantly improve LBP and disability in patients with CLBP and MCs, their improvements may not be clinically significant or safe. To date, no relevant systematic reviews have been conducted to summarize the evidence, and these important questions remain unanswered. Therefore, this systematic review and meta‐analysis aimed to summarize the evidence regarding the effectiveness and safety of oral antibiotic use for patients with CLBP who exhibit MCs on MRI.

## 2 MATERIALS AND METHODS

The study was conducted and reported according to the Preferred Reporting Items of Systematic Reviews and Meta‐analyses (PRISMA) guidelines. The protocol was registered on PROSPERO (CRD42021219667).

### 2.1 Search strategy

MEDLINE, EMBASE, CINAHL, Allied and Complementary Medicine, and Cochrane Library were searched from inception to March 3, 2023. There was no language restriction in the search. The search string included the combinations of keywords and medical subject headings (MeSH) related to (1) patients with CLBP aged 18 years or older; (2) disc degeneration, MCs, or endplate signal changes; (3) RCTs or non‐RCTs; and (4) antibiotics. The search strategies/definitions are illustrated in Tables A1, A2, A3, A4, A5. The reference lists of all included studies were screened for relevant articles. The corresponding authors of the included studies were contacted by email to identify additional relevant publications or relevant data to facilitate meta‐analyses. Forward citation tracing was conducted using Scopus.

### 2.2 Selection criteria

Studies were included if they investigated the effectiveness of antibiotics in patients with CLBP (aged 18 years or older) with or without disc degeneration‐related surgery. The definition of CLBP might vary slightly across studies. Both RCTs and non‐RCTs studies were eligible for inclusion to obtain a comprehensive overview of this topic. MRI‐confirmed lumbar‐related MCs should be reported in the included studies. Studies were excluded if they involved acute hematogenous osteomyelitis, or spondylodiscitis related to *Staphylococcus aureus* (e.g., *methicillin*‐*susceptible Staphylococcus aureus bacteriemia*, *methicillin*‐*resistant Staphylococcus aureus bacteriemia*), or high virulent infection. Additionally, case reports, letters to the editor, conference proceedings, and commentaries were excluded.

### 2.3 Study selection

Citations identified from databases were exported to EndNote X9.2 (Thomson Reuters). After removing duplicates, two independent reviewers (G.M. and A.W.) independently screened the titles and abstracts of potential citations according to the selection criteria. Between‐reviewers' disagreements were discussed to obtain consensus. Any remaining disagreement was resolved by a third reviewer (D.S.). Relevant systematic/literature reviews were included for full‐text reading to identify additional primary studies for screening. The full‐text screening procedure was identical to the abstract screening.

### 2.4 Data extraction

Relevant data from the included studies were independently extracted and counterchecked by the two reviewers (G.M. and A.W.). The extracted data included: authors' names, year of publication, study design, study location, settings, study objectives, response and/or attrition rates, the randomization method (if applicable), participants' selection criteria, definitions of CLBP, types of medical imaging, types of MCs, oral antibiotics and/or other control treatments, follow‐up duration and numbers, primary and secondary outcomes, relevant statistics, reported clinical significance, and side effects.

### 2.5 Risk of bias assessments

Two independent reviewers (S.P. and A.W.) evaluated the risk of bias in the included studies. Specifically, the risk of bias of the included RCTs was evaluated by the Risk of Bias 2 (RoB 2) tool. Because only relevant case series were identified, the included case series was evaluated by the National Institutes of Health quality assessment tool for before‐after studies with no control group. The two reviewers' assessments were compared. Any discrepancies were resolved by consensus.

### 2.6 Data synthesis

If the primary or secondary outcome measures were comparable between two or more included studies, relevant data were pooled for meta‐analyses using RevMan v.5.3 (The Cochrane Collaboration, Software Update, Oxford, UK). If the between‐group differences in clinical outcomes of a primary study were reported as medians and interquartile ranges, these values were converted to means and standard deviations using the McGrath et al. method to enable meta‐analyses.^32^ The pooled between‐group differences in outcome measures were reported as mean differences (MDs) and 95% confidence intervals (95% CIs). The forest plot and *I* ^2^ statistics of each pooled outcome measure were reported. Random effects models were used for meta‐analyses with *I* ^2^ > 50% to account for differences across heterogeneous studies.^33^, ^34^ If *I* ^2^ < 50%, fixed effects models were used. If there were ≥10 studies in a given meta‐analysis, the publication bias was assessed by a funnel plot.^35^ The alpha level was set at 0.05 for all meta‐analyses. If a given clinical outcome could not be meta‐analyzed, the finding was summarized narratively. Sensitivity analyses were conducted to evaluate the results after eliminating low‐quality studies.

### 2.7 The GRADE evaluation

Two authors (S.P. and A.W.) independently assessed the quality of evidence regarding the effect of oral antibiotics on each reported clinical outcome using the Grading of Recommendations Assessment, Development and Evaluation (GRADE) http://gradepro.org. The quality of evidence was rated as high, moderate, low, or very low.

## 3 RESULTS

**FIGURE 1:**
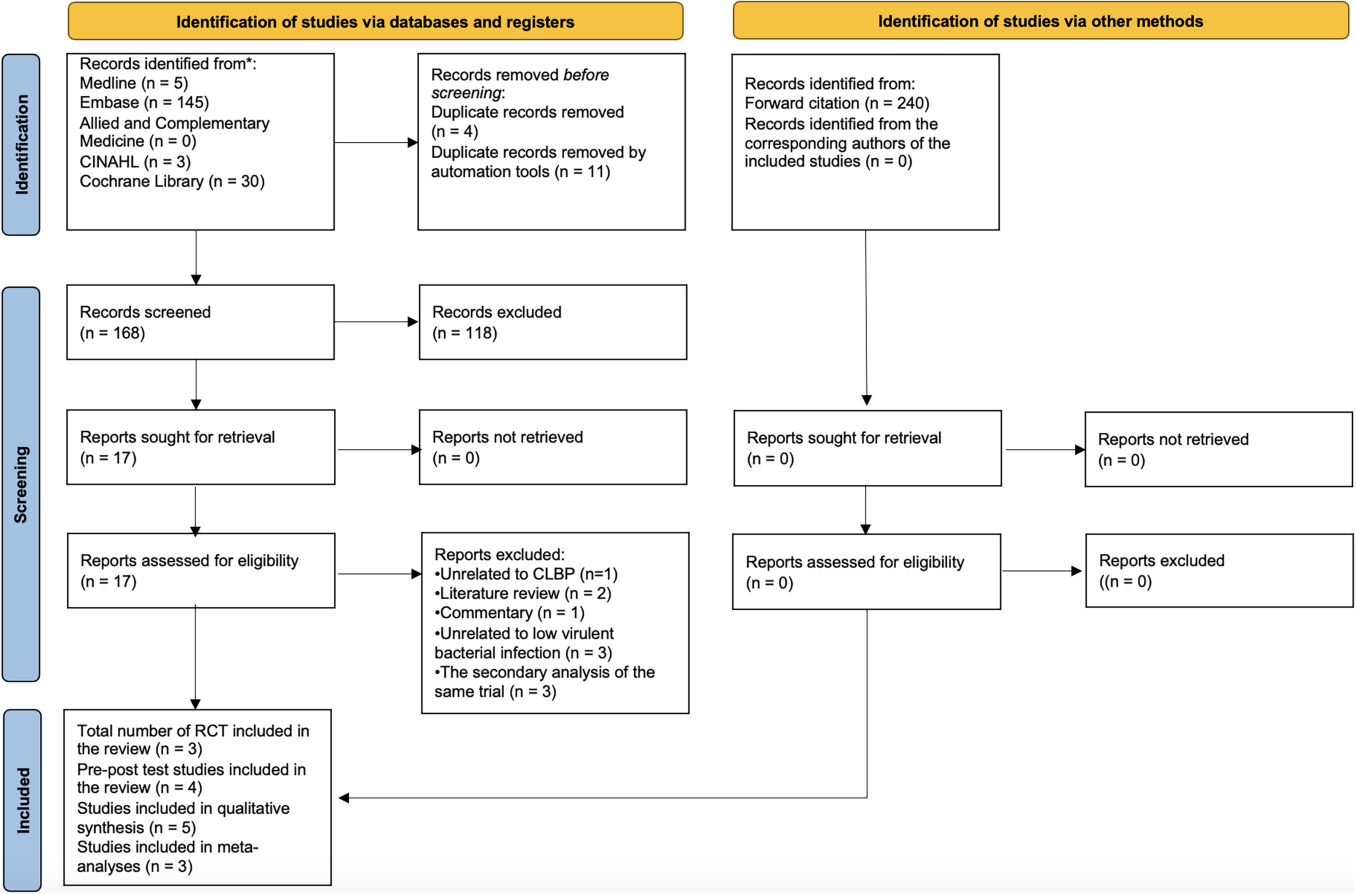
A flow diagram of the systematic review.

The systematic searches and forward citations yielded 183 and 240 citations, respectively. After removing duplicates, 408 titles and abstracts were screened. Fourteen were included for full‐text screening. Seven full‐text articles (three RCTs^28^, ^29^, ^36^ and four case series^37^, ^38^, ^39^, ^40^) involving 1508 people with CLBP and concomitant MCs were included (Figure 1). These studies were conducted in Canada, Denmark, France, Iraq, and Norway. Five included cohorts involved patients with CLBP and MC1, while two included cohorts involved patients with CLBP and MC1 and MC2, and/or mixed MCs.^29^, ^37^ Amoxicillin (750 mg) three times per day for 90 days and Amoxicillin‐clavulanate (500 mg/125 mg) three times per day for 100 days were used in two and five included studies, respectively, while calcium carbonate or maize starch was used in the placebo pills of the included RCTs (Table 1). All included studies were followed up immediately post‐treatment, or at 11, 12, or 24 months after the treatment commencement. Because no meta‐analysis involved 10 or more articles, no publication bias was analyzed.

**TABLE 1:** Characteristics of the included studies.

Authors/year of publication	Country/Patient characteristics/Initial sample size/No. of completed participants/Mean age (SD)/Percentage of male	Sample methods	Randomization method/response rate/attrition rate (if applicable)	Treatment/treatment duration	Measurement time points/side effects of antibiotics/limitations	Sample size calculation/Statistical tests/outcome measures
*Randomized controlled trials*						
Albert et al., 2013	Denmark/double‐blinded (assessor and patients) RCT/162 patients with LBP > 6 months + new MC1/147 completed 1‐year FU with questionnaires 144 completed 1‐year FU with MRI + physical exam/Antibiotics: 44.7 (10.3) years; 58.2% male Placebo: 45.5 (9.2) years; 58.2% male	Consecutive sample from two secondary spine centers by invitation letters. Aged: 18–65 years Initial MRI: disc herniation L3/4, L4/5, or L5/S1 in the last 6–24 months LBP > 6 months; LBP rating scale (0–30)/3: ≥ 6 Surgically/nonsurgically treated Repeated MRI: MC1 adjacent to the previously herniated disc	Randomized by the separate center using a computer‐generated randomization list and retained until 1‐year FU. Attrition: Antibiotics: 14.4% Placebo: 6.9%	Gp1: 1 Amoxicillin‐clavulanate (500 mg/125 mg) (Bioclavid®) pill, 3 times/day for 100 days/(*n* = 45) Gp2: 2 (Bioclavid®) (500 mg/125 mg) pills, 3 times/day for 100 days/(*n* = 45) Gp3: 1 calcium carbonate (placebo) pill, 3 times/day/100 days/(*n* = 36) Gp4: 2 placebo pills 3 times/day/100 days/(*n* = 36) Recommended no other treatment or exercise (except mild analgesics and anti‐inflammatory medication) during the 1‐year period but all other treatments were documented at FU.	Baseline, 100‐day FU, 1‐year FU for questionnaires Baseline, 1‐year FU for blood sampling and MRI Side effects: low‐grade gastroenterological complaints (e.g., loose bowel movements, increased flatus, or burping); middle‐grade gastroenterological complaints >3 months Limitations: No post‐treatment MRI scans; no biopsy, heterogenous sample, Bang blinding index of 0.52 in the placebo group (some unblinding of the group), some patients in the placebo group might take amoxicillin but all medical records have been cross‐checked.	Calculated sample size: 65/group based on two‐group comparison between antibiotics and placebo but the Danish Medical authorities had a late request for a dose–response investigation (which had no sample size calculation) ITT: For binary variables: Fisher's exact test; For continuous variables: Crude independent *t* tests, between‐group comparison adjusted for age and gender; Mann–Whitney test for unnormalized data. No interim analysis Primary: RMDQ, LBP Secondary: Leg pain No. of hours with pain in the last 4 weeks Global perceived health, EQ‐5D thermometer Days with sick leave Bothersomeness Constant pain 4 physical tests (Valsalva maneuver, active flexion/extension, springing test, cranial compression test) Serum analysis results MRI findings Clinical significant difference: > 30% reduction in baseline RMDQ score, and 2 LBP rating scale points
Al‐Falahi et al., 2014	Unclear blinding 71 patients with LBP > 6 months + MC1/ Antibiotics: 49.4 years; 50% male Placebo: 48.9 years; 58% male	Patients with LBP > 6 months after a prior disc herniation + MC1 Consecutive sample from an orthopedic department of a hospital, participants should be aged: 18–65 years MC1 adjacent to a prior disc herniation L2/3, L3/4, L4/5, or L5/S1 within the last 6–24 months LBP > 6 months; LBP rating scale (0–30)/3: ≥5 Surgically/nonsurgically treated	Unclear randomization method Attrition: Antibiotics: 38.1% Placebo: 41.4% (young people tended to quit)	Gp1: 1 Amoxicillin‐clavulanate (500 mg/125 mg) (Gloclav®) pill, 2 times/day for 100 days/(*n* = 42) Gp2: 1 calcium carbonate (500 mg) (Calcid) (placebo) pill, 3 times/day/100 days/(*n* = 29) No other treatment except mild analgesics (paracetamol)	Baseline, 100‐day FU Side effects of antibiotics: low‐grade gastroenterological complaints (e.g., loose bowel movements, increased flatus or infrequent attacks of abdominal colic)	No sample size calculation Unknown statistical analysis Outcomes RMDQ LBP rating scale Physical exams Blood sampling at baseline Checking for medication compliance, and side effect, and other concomitant medications Clinically significant difference: >30% reduction in baseline RMDQ score, and 2 LBP rating scale points
Bråten et al., 2019	Norway/double‐blinded (participants, statistician, care providers, research staff), parallel‐group RCT; 180 patients with LBP > 6 months, with MC1 (*n* = 118) or MC2 (*n* = 62), at the level of previous disc herniation Antibiotics: 44.7 (9.0) years; 47% male Placebo: 45.2 (9.0) years; 48% male	Consecutive sample from outpatient clinics at 6 hospitals Patients with LBP > 6 months Aged: 18–65 years Lumbar disc herniation in the last 24 months Baseline 1.5 T MRI scan: showing MC1 or MC2 (≥10% of vertebral height and diameter > 5 mm) at the level adjacent to the previously herniated disc LBP > 6 months; LBP rating scale (0–30)/3: ≥5 Nonsurgically treated Patients only; Without antibiotic treatment in the last month	Block randomization (1:1:1:1) for MC1, MC2, previous surgery and without surgery) with random block size of 4 or 6. Participants were randomized within 13 days after inclusion by the separate center using a computer‐generated random number	Gp1: 1 Amoxicillin (750 mg) pill, 3 times/day for 3 months/(*n* = 89) Gp2: 1 maize starch pill encapsulated (Kragerø Tablettproduksjon AS) (placebo) tablet, 3 times/day/for 3 months/(*n* = 91) Recommended not to start additional LBP treatments or use nonsteroid anti‐inflammatory drugs but allowed to continue ongoing treatments (except mild analgesics and anti‐inflammatory medication) during the 1‐year period but all treatments were documented.	Baseline, 13‐week FU, 12‐month FU Adverse events were checked by clinical patient notes Adverse events of antibiotics: Abdominal pain, diarrhea, rash, vaginal Candida infection, oral Candida infection Limitations Costs of antibiotic resistance or adverse events were not considered; no biopsy to confirm the presence of bacteria.	Calculated sample size: 66/group for assessing MC1 and MC2 subgroups. ITT analysis and per protocol analyses For primary analysis, analysis of covariance (ANCOVA) with adjustment of baseline RMDQ score and the stratification variables; Missing RMDQ data was imputed by a multiple imputation model For secondary analyses, separate ANCOVA for RMDQ of two MC types. Other secondary outcomes were also analyzed. Other sensitivity analyses; responder analyses based on >30%, >50%, and >75% improvement from baseline, excluding patients with >30% missing RMDQ items; linear mixed effect models With interim analysis Primary: RMDQ at 1‐yr FU Secondary: ODI NPRS EQ5D‐5L Weekly patient‐reported questionnaires for compliance Counting the number of returned study drug capsules at the end of treatment visit Band blinding Index Clinically significant difference: reduction of baseline Norwegian RMDQ by 4 points, ODI by 13–20 points; NPRS by 2–3 points; EQ5D‐5L by 0.11–0.30
Case series						
Albert et al., 2008	Denmark/32 patients with LBP and MC1 after adjacent lumbar disc herniation/Male (*n* = 10); 47.7 (8.2) years/Female (*n* = 10); 45.7 (11.1) years	Patients with LBP and MC1 at 14‐month FU of an RCT that investigated 2 active treatments for Lx herniated disc were waited for another 9 months before participating in this study	9.4% dropped out due to severe diarrhea.	Amoxicillin‐clavulanate (500 mg/125 mg) three times per day with optional mild analgesics/90 days	Baseline, end of treatment, 11‐month FU Severe diarrhea	Wilcoxon signed‐rank tests Outcomes: LBP intensity, no. of days with pain, disease RMDQ, global perceived effect, Self‐perceived function scale, serum analysis Limitations: No post‐treatment MRI scans; no biopsy observational study with a highly selected group of patients
Albert et al., 2017	Denmark/1024 patients with LBP and MCI after lumbar disc herniation	Consecutive sample from a clinic Patients with LBP >6 months Aged: 18–65 years Surgically or nonsurgically treated patients MRI: MC1, MC2, or mixed MC in the vertebra adjacent to a previous disc herniation	3.6% dropped out after 100 days of treatment 41.2% dropped out at the 12‐month follow‐up; 73.6% dropped out at the 24‐month follow‐up.	Two Amoxicillin‐clavulanate (500 mg/125 mg) (Bioclavid®) pills, three times/day for 100 days/(*n* = 42) Patients could continue their usual treatments (e.g., anti‐inflammatory drugs, or pain relief drugs) but not to do exercise	Baseline, end of treatment, 12‐month FU, 24‐month FU	No sample size calculation Unknown statistical analysis Outcomes RMDQ LBP rating scale NPRS EQ5D‐5L Units of analgesia Clinically significant difference: >30% reduction in baseline RMDQ score, and 2 LBP rating scale points
Gupta et al., 2017	Canada/11 patients with LBP and MC 40.6 ± 3.2 years/81.8% males	Research from three clinics Patients with LBP >6 months Aged: 18–70 years MRI in the last year: MC1 and related herniated disc at least 1 level. If mixed MC or multiple levels were affected, MC1 should be predominated. Poor response to physiotherapy and anti‐inflammatory treatment for at least 3 months	No attrition.	Amoxicillin‐clavulanate (500 mg/125 mg) three times per day for at least 90 days/with optional mild analgesics/Patients could continue doing exercises, physiotherapy, spinal manipulation, medication treatment	Baseline, end of treatment, 12‐month FU Side effects: mild gastrointestinal symptoms (e.g., loose bowel movements, increased flatus, or burping)	No sample size calculation Unknown statistical analysis Outcomes ODI NPRS Repeated MRI scan after 1 year Minimally important clinical difference: ≥ 30% reduction in NPRS and/or a 20% improvement in ODI
Palazzo et al., 2017	28 patients with LBP and MC1/46.6 (range: 34–57) years/42.9% males	Consecutive sampling of patients with chronic LBP for >6 months MRI showed: MC1 at L3/4, L4/5, and L5/S1 who failed usual conservative treatments	Drop out 28.6% due to severe side effects or no improvement	Amoxicillin‐clavulanate (500 mg/125 mg) three times per day for at least 100 days Probiotics were used to prevent diarrhea Analgesics, anti‐inflammatory drugs, spinal steroid injection, bracing, and physiotherapy were allowed	Baseline, end of treatment, 12‐month FU for MRI on 12 participants Adverse effects: reported but no details	No sample size calculation Unknown statistical analysis Outcomes NPRS Global improvement Morning stiffness No. of night‐time awakening Analgesics consumption Repeated MRI between 6 and 12 months for 12 participants

Abbreviations: EQ5D‐5L, EuroQol's health‐related quality of life, version 2; NPRS, 11‐point numeric pain rating scale.

### 3.1 Risk of bias assessments

**FIGURE 2:**
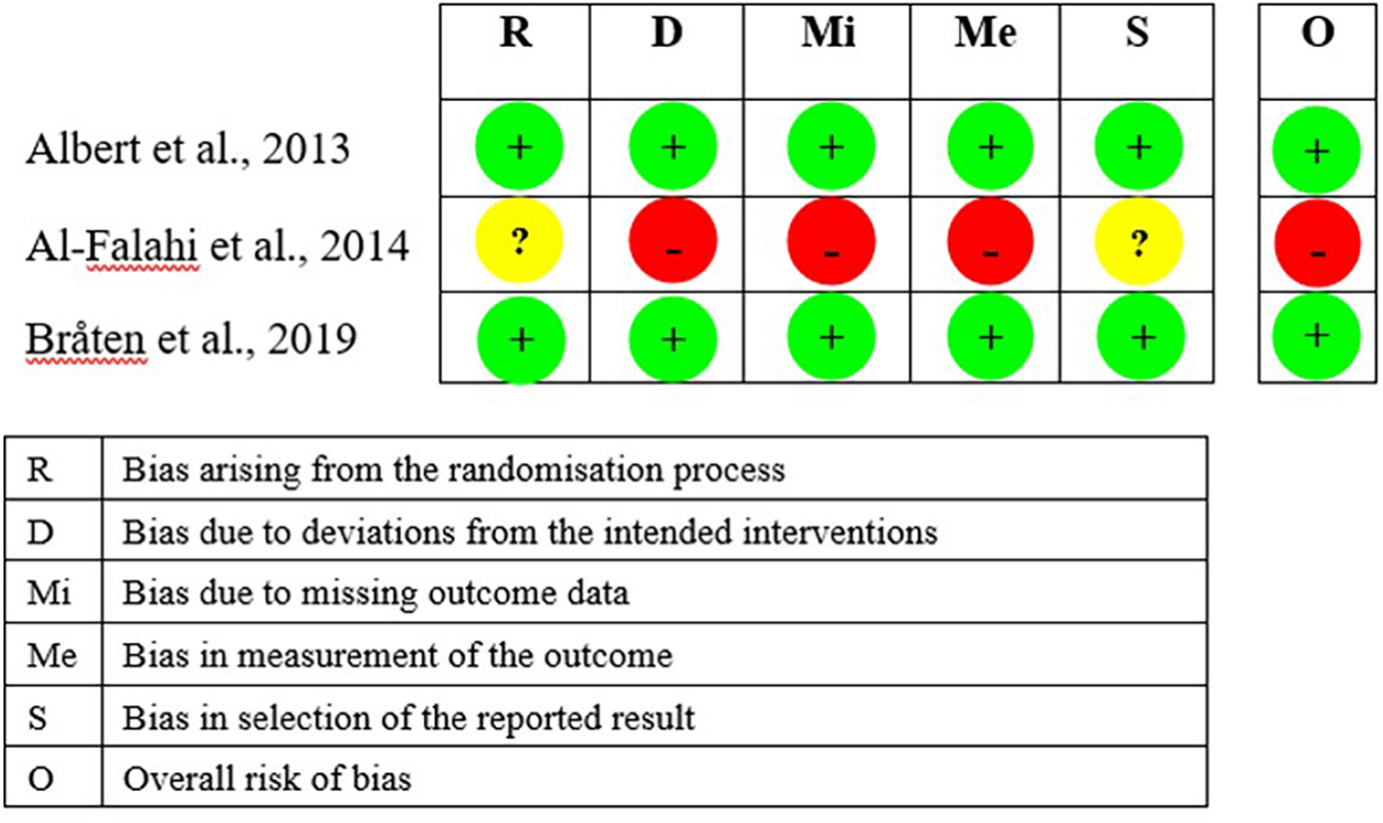
Risk of bias assessments of the included randomized controlled trials. “+” = low risk of bias; “–” = high risk of bias; “?” = some concerns.

Two of the three included RCTs had a low risk of bias (Figure 2). Although one RCT had a low risk of bias, its statistical analysis plan was not described in the research protocol on a clinical trial registry.^28^ The RCT with a high risk of bias had an unclear randomization procedure, no information regarding the blinding of clinicians or assessors, high attrition rate (>38%), no information about whether the statistical analyses followed the predetermined plan (Figure 2).^36^ The four case series had fair or poor quality (Table 2). Their common sources of bias were unclear eligibility criteria, no sample size calculation, unclear blinding of assessors, and unknown reasons for loss to follow‐up.

**TABLE 2:** NIH quality assessment for before–after (pre–post) studies with no control group.

	Q1	Q2	Q3	Q4	Q5	Q6	Q7	Q8	Q9	Q10	Q11	Q12	Total score	Quality rating
Albert et al., 2008	Y	N	CD	NR	NR	Y	Y	NR	Y	Y	Y	NA	6/12	Fair
Albert et al., 2017	Y	Y	Y	NR	Y	Y	Y	NR	N	Y	Y	NA	8/12	Fair
Gupta et al., 2017	Y	Y	Y	NR	N	Y	Y	NR	Y	N	Y	Y	8/12	Fair
Palazzo et al., 2017	N	N	Y	NR	N	Y	N	NR	N	Y	Y	NA	4/12	Poor

*Note*: Q1: Was the study question or objective clearly stated? Q2: Were eligibility/selection criteria for the study population prespecified and clearly described? Q3: Were the participants in the study representative of those who would be eligible for the test/service/intervention in the general or clinical population of interest? Q4: Were all eligible participants that met the prespecified entry criteria enrolled? Q5: Was the sample size sufficiently large to provide confidence in the findings? Q6: Was the test/service/intervention clearly described and delivered consistently across the study population? Q7: Were the outcome measures prespecified, clearly defined, valid, reliable, and assessed consistently across all study participants? Q8: Were the people assessing the outcomes blinded to the participants' exposures/interventions? Q9: Was the loss to follow‐up after baseline 20% or less? Were those lost to follow‐up accounted for in the analysis? Q10: Did the statistical methods examine changes in outcome measures from before to after the intervention? Were statistical tests done that provided p values for the pre‐to‐post changes? Q11: Were outcome measures of interest taken multiple times before the intervention and multiple times after the intervention (i.e., did they use an interrupted time‐series design)? Q12: If the intervention was conducted at a group level (e.g., a whole hospital, a community, etc.) did the statistical analysis take into account the use of individual‐level data to determine effects at the group level? Total Score: Number of yes; CD, cannot be determined; NA, not applicable; NR, not reported; N, no; Y, yes.

### 3.2 Between‐group comparisons in pain reduction among patients with CLBP and/or leg pain

**FIGURE 3:**
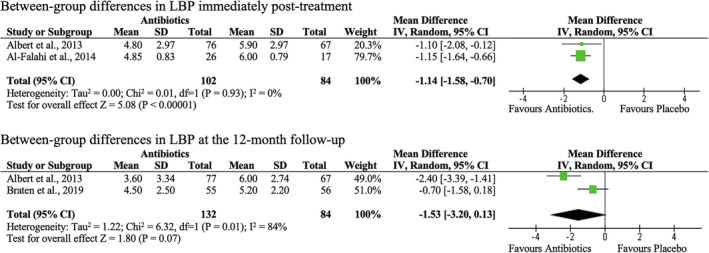
Forest plots of effects of antibiotics versus placebo on low back pain (LBP) intensity in patients with chronic low back pain and type 1 Modic changes immediately post‐treatment or at the 12‐month follow‐up (9‐month post‐treatment).

Two meta‐analyses were conducted to evaluate the effectiveness of oral antibiotics in improving LBP in people with CLBP and MC1 immediately after treatment or at the 12‐month follow‐up (Figure 3). Low‐quality evidence from two RCTs supported that Amoxicillin‐clavulanate was significantly better than placebo pills in reducing LBP immediately after 100 days of treatments in CLBP patients with MC1 (MD: −1.14; 95% CI: −1.58 to −0.70; *I* ^2^ = 0%; Figure 3), which was not clinically significant according to the reported minimal clinically important difference for LBP intensity.^41^ Low‐quality evidence from another meta‐analysis substantiated that oral antibiotic was not significantly better than placebo in reducing LBP in patients with CLBP at the 12‐month follow‐up (Figure 3). However, low‐quality evidence from a RCT suggested that compared to CLBP patients with MC1 in the antibiotic group, a significantly greater proportion of patients in the placebo group had constant back pain, LBP‐related sleep disturbance, positive cranial compression test, and pain during lumbar flexion or extension, or Valsalva maneuver at the 12‐month follow‐up (Table 3, Table A2).^28^ The same study found that patients in the antibiotic group had significantly less hours with LBP in the last 28 days than those in the placebo group at the 12‐month follow‐up.^28^ As expected, low‐quality evidence showed that Amoxicillin was not significantly better than placebo in improving LBP in patients with MC2 alone, or mixed MCs immediately post‐treatment or at the 12‐month follow‐up (Table A2).

**TABLE 3:** The effects of antibiotics versus placebo on clinical outcomes among people with chronic low back pain in the included randomized controlled trials.

	Study (initial sample size), study design	Measurement tools	Baseline	End of treatment	1‐year follow‐up	2‐year follow‐up	*p*‐value	Effect (Pooled)	Strength of evidence
*Low back pain*									
LBP	*Between*‐*group comparisons for patients with MC1 only*								
	Albert et al., 2013 (Amoxicillin‐clavulanate: *n* = 90) (Placebo: *n* = 72) RCT	LBP rating scale [(current + (worst + mean in the last 2 weeks)/3) (0 is the best) (MC1 only)	Amoxicillin‐clavulanate: 6.7 (5.3 and 7.7) (*n* = 90) Placebo: 6.3 (4.7 and 8.0) (*n* = 72) Median (25th and 75th percentile)	Amoxicillin‐clavulanate: 5.0 (2.7 and 6.7) (*n* = 76) Placebo: 6.3 (3.7 and 7.7) (*n* = 67) Median (25th and 75th percentile)	NA	NA	Not reported for between‐group and within‐group comparisons	Between‐group difference with reference to the placebo group: −1.14 (−1.58 to −0.0.70) *p* <0.00001; *I* ^2^ = 0%	Low
	Al‐Falahi et al., 2014 (Amoxicillin‐clavulanate: *n* = 42) (Placebo; *n* = 29)	LBP rating scale [(current + (worst + mean in the last 2 weeks)/3) (0 is the best) (MC1 only)	Amoxicillin‐clavulanate: 6.42 (0.90) (*n* = 26) Placebo: 6.12 (0.86) (*n* = 17) Mean (SD)	Amoxicillin‐clavulanate: 4.85 (0.83) (*n* = 26) Placebo: 6.00 (0.79) (*n* = 17) Mean (SD)	NA	NA	*p* = 0.001 for within‐group antibiotics only *p* <0.05 for between‐group comparisons		
	Albert et al., 2013 (Amoxicillin‐clavulanate: *n* = 90) (placebo: *n* = 72) RCT	LBP rating scale [(current + (worst + mean in the last 2 weeks)/3) (0 is the best) (MC1 only)	Amoxicillin‐clavulanate: 6.7 (5.3–7.7) (*n* = 90) Placebo: 6.3 (4.7 and 8.0) (*n* = 72) Median (25th and 75th percentile)		At 12‐month FU: Amoxicillin‐clavulanate: 3.7 (1.3–5.8) (*n* = 77) Placebo: 6.3 (4 and 7.7) (*n* = 67) Median (25th and 75th percentile)	NA	*p* <0.001 between antibiotics and placebo at 1‐year FU	Between‐group difference with reference to the placebo group: −1.53 (−3.20 to 0.13) *p* = 0.07; *I* ^2^ = 84%	Low
	Bråten et al., 2019 (Amoxicillin: *n* = 89) (Placebo; *n* = 90)	NPRS last week (MC1 only) NPRS between‐group differences (95% CI) (MC1 only)	Antibiotics: 6.5 (1.1) (*N* = 58) Placebo: 6.3 (1.3) (*N* = 59) Mean (SD)		Antibiotics: 4.5 (2.5) (*n* = 55) Placebo: 5.2 (2.2) (*n* = 56) −0.8 (−1.6 to 0.0) (*n* = 117) Mean (SD)	NA	*p* = 0.06		
	*Between*‐*group comparisons for patients with MC2 only*								
	Bråten et al., 2019 (Amoxicillin: *n* = 30) (Placebo; *n* = 31)	NPRS (MC2 only) NPRS between‐group differences (95% CI) (MC2 only)	Antibiotics: 6.3 (1.3) (*N* = 30) Placebo: 6.2 (1.9) (*N* = 31) Mean (SD)	NA	Antibiotics: 5.0 (1.9) (*n* = 30) Placebo: 5.2 (2.5) (*n* = 28) −0.3 (−1.3 to 0.7) (*n* = 62) Mean (SD)	NA	*p* = 0.52		Low
	*Between*‐*group comparisons for patients with MC1 and MC2*								
	Bråten et al., 2019 (Amoxicillin: *n* = 89) (Placebo; *n* = 90)	NPRS last week (Both MC1 and MC2) NPRS between‐group differences (95% CI) (Both MC1 and MC2)	Antibiotics: 6.4 (1.2) (*N* = 88) Placebo: 6.3 (1.5) (*N* = 90) Mean (SD)	Antibiotics: 5.2 (2.3) (*n* = 85) Placebo: 5.4 (1.9) (*n* = 85) −0.6 (−1.3 to 0.0) (*n* = 179) Mean (SD)	NA	NA	*p* = 0.33 between‐groups		Low
	Bråten et al., 2019 (Amoxicillin: *n* = 89) (Placebo; *n* = 90)	NPRS (Both MC1 and MC2) NPRS between‐group differences (95% CI) (Both MC1 and MC2)	Antibiotics: 6.4 (1.2) (*N* = 88) Placebo: 6.3 (1.5) (*N* = 90) Mean (SD)	NA	Antibiotics: 4.7 (2.3) (*n* = 85) Placebo: 5.2 (2.3) (*n* = 85) −0.6 (−1.3 to 0.0) (*n* = 179) Mean (SD)	NA	*p* = 0.06		Low
*Between*‐*group comparisons at the 1*‐*year follow*‐*up for patients with MC1 only*									
Proportion of patients with symptoms	Albert et al., 2013 (Amoxicillin‐clavulanate: *n* = 90) (Placebo: *n* = 72)	The presence of LBP (MC1 only)	Antibiotics: 100.0% Placebo: 100.0%	NA	Antibiotics: 67.5% (*n* = 77) Placebo: 94.0% (*n* = 67)	NA	Between‐group difference *p* = 0.0001		Low
		Had constant pain (MC1 only)	Antibiotics: 75.3% Placebo: 73.1%	NA	Antibiotics: 19.5% (*n* = 77) Placebo: 67.2% (*n* = 67)	NA	Between‐group difference *p* = 0.0001		Low
		Disturbed sleep at night due to pain (MC1 only)	Antibiotics: 74.0% Placebo: 76.1%	NA	Antibiotics: 29.9% (*n* = 77) Placebo: 61.2% (*n* = 67)	NA	Between‐group difference *p* = 0.001		Low
		Had pain during the Valsalva maneuver (MC1 only)	Antibiotics: 75.3% Placebo: 71.6%	NA	Antibiotics: 41.6% (*n* = 77) Placebo: 56.7% (*n* = 67)	NA	Between‐group difference *p* = 0.05		Low
		Had pain during active lumbar flexion (MC1 only)	Antibiotics: 96.1% Placebo: 100.0%	NA	Antibiotics: 49.4% (*n* = 77) Placebo: 83.6% (*n* = 67)	NA	Between‐group difference *p* = 0.0001		Low
		Had pain during active lumbar extension (MC1 only)	Antibiotics: 87.0% Placebo: 86.6%	NA	Antibiotics: 51.9% (*n* = 77) Placebo: 74.6% (*n* = 67)	NA	Between‐group difference *p* = 0.0001		Low
		Had pain during springing test (MC1 only)	Antibiotics: 92.2% Placebo: 94.0%	NA	Antibiotics: 55.8% (*n* = 77) Placebo: 77.6% (*n* = 67)	NA	Between‐group difference *p* = 0.006		Low
		Positive cranial compression test (MC1 only)	Antibiotics: 36.4% Placebo: 35.8%	NA	Antibiotics: 19.5% (*n* = 77) Placebo: 34.3% (*n* = 67)	NA	Between‐group difference *p* = 0.044		Low
*Between*‐*group clinically significant changes in LBP*									
LBP	Al‐Falahi et al., 2014 (Amoxicillin: *n* = 42) (Placebo; *n* = 29)	At least two‐point decrease in LBP rating scores (MC1 only)	NA	Antibiotics (10 out of 26) Placebo: (2 out of 17)	NA	NA	*p* = 0.001 (for within‐group antibiotics only)		Very low
*Between*‐*group changes in pain duration immediate post*‐*treatment and at the 1*‐*year follow*‐*up for patients with MC1*									
Number of hours with LBP in the last 28 days	Albert et al., 2013 (Amoxicillin‐clavulanate: *n* = 90) (Placebo: *n* = 72)	Total hours of LBP in the last 28 days (MC1 only)	Antibiotics: 448 (364 and 448) (*n* = 90) Placebo: 448 (392 and 448) (*n* = 72) Median (25th and 75th percentile)	Antibiotics: 180 (16 and 136) (*n* = 76) Placebo: 200 (28 and 392) (*n* = 67) Median (25th and 75th percentile)	Antibiotics: 64 (4 and 280) (*n* = 77) Placebo: 448 (224 and 448) (*n* = 67) Median (25th and 75th percentile)	NA	*p* = 0.0001 at the 1‐year FU		Low
**Leg pain**									
*Between*‐*group comparisons*									
Leg pain	*Patients with MC1 only*								
	Albert et al., 2013 (Amoxicillin‐clavulanate: *n* = 90) (Placebo: *n* = 72)	11‐point NPRS (MC1 only)	Antibiotics: 5.3 (2.3 and 7) (*n* = 90) Placebo: 4.0 (1 and 7) (*n* = 72) Median (25th and 75th percentile)	Antibiotics: 3.0 (1 and 5.7) (*n* = 76) Placebo: 4.3 (1 and 7) (*n* = 67) Median (25th and 75th percentile)	Antibiotics: 1.7 (0 and 4.2) (*n* = 77) Placebo: 4.3 (1 and 6.3) (*n* = 67) Median (25th and 75th percentile)	NA	Between‐group difference at 1‐year FU *p* = 0.004		Low
	*Patients with MC1 and MC2*								
	Bråten et al., 2019 (Amoxicillin: *n* = 89) (Placebo; *n* = 90)	NPRS (Both MC1 and MC2) Between‐group differences (95% CI) (Both MC1 and MC2)	Antibiotics: 3.2 (2.6) (*n* = 89) Placebo: 3.2 (2.6) (*n* = 90) Mean (SD)	Antibiotics: 3.1 (2.8) (*n* = 85) Placebo: 3.4 (2.6) (*n* = 84) Mean (SD)	Antibiotics: 2.8 (2.7) (*n* = 85) Placebo: 3.5 (2.8) (*n* = 82) −0.8 (−1.4 to −0.1) (*n* = 166) Mean (SD)	NA	Between‐group difference at 1‐year FU *p* = 0.03		Low
**LBP‐related disability**									
*Between*‐*group comparisons*									
LBP‐related disability	*Patients with MC1 only*								
	Albert et al., 2013 (Amoxicillin‐clavulanate: *n* = 90) (Placebo: *n* = 72)	RMDQ (MC1 only)	Antibiotics: 15 (11 and 18) (*n* = 90) Placebo: 15 (12 and 18) (*n* = 72) Median (25th and 75th percentile)	Antibiotics: 11.5 (7 and 14) (*n* = 76) Placebo: 14 (11 and 18) (*n* = 67) Median (25th and 75th percentile)	NA	NA	Not reported	Between‐group difference with reference to the placebo group: −3.28 (−4.61 to −1.96) *p* <0.00001; *I* ^2^ = 44%	Low
	Al‐Falahi et al., 2014 (Amoxicillin‐clavulanate: *n* = 42) (Placebo; *n* = 29)	RMDQ (MC1 only)	Antibiotics: 15.50 (1.66) (*n* = 26) Placebo: 15.00 (1.70) (*n* = 17) Mean (SD)	Antibiotics: 12.04 (1.93) (*n* = 26) Placebo: 14.82 (1.47) (*n* = 17) Mean (SD)	NA	NA	*p* < 0.05		
	Albert et al., 2013 (Amoxicillin‐clavulanate: *n* = 90) (Placebo: *n* = 72)	RMDQ (MC1 only)	Antibiotics: 15 (11 and 18) (*n* = 90) Placebo: 15 (12 and 18) (*n* = 72) Median (25th and 75th percentile)	NA	At 1‐year FU: Antibiotics: 7.0 (4 and 11) (*n* = 77) Placebo: 14 (8 and 18) (*n* = 67) Median (25th and 75th percentile)	NA	Between‐group difference at 1‐year FU *p* < 0.001	Between‐group difference with reference to the placebo group: −3.71 (−6.86 to −0.55) *p* = 0.02; *I* ^2^ = 77%	Moderate
	Bråten et al., 2019 (Amoxicillin: *n* = 89) (Placebo; *n* = 91)	RMDQ (MC1 only) RMDQ between‐group differences (95% CI) (MC1 only)	Antibiotics: 12.9 (4.3) (*n* = 58) Placebo: 12.3 (3.7) (*n* = 60) Mean (SD)	NA	Antibiotics: 8.2 (6.0) (*n* = 55) Placebo: 10.3 (5.4) (*n* = 56) −2.3 (−4.2 to −0.4) (*n* = 118) Mean (SD)	NA	*p* = 0.02		
	Bråten et al., 2019 (Amoxicillin: *n* = 89) (Placebo; *n* = 91)	ODI (MC1 only) ODI between‐group differences (95% CI) (MC1 only)	Antibiotics: 31.3 (11.5) (*n* = 58) Placebo: 30.4 (9.9) (*n* = 58) Mean (SD)	NA	Antibiotics: 23.4 (15.2) (*n* = 55) Placebo: 27.7 (13.8) (*n* = 56) −5.1 (−9.3 to −0.8) (*n* = 117) Mean (SD)	NA	Between‐group *p* = 0.02		Low
	*Patients with MC2 only*								
	Bråten et al., 2019 (Amoxicillin: *n* = 89) (Placebo; *n* = 91)	RMDQ (MC2 only) RMDQ between‐group differences (95% CI) (MC2 only)	Antibiotics: 12.3 (5.5) (*n* = 30) Placebo: 13.7 (3.5) (*n* = 30) Mean (SD)	NA	Antibiotics: 10.5 (6.5) (*n* = 30) Placebo: 11.4 (5.9) (*n* = 28) −0.1 (−2.7 to 2.6) (*n* = 62) Mean (SD)	NA	*p* = 0.95		Low
	Bråten et al., 2019 (Amoxicillin: *n* = 89) (Placebo; *n* = 91)	ODI (MC2 only) ODI between‐group differences (95% CI) (MC2 only)	Antibiotics: 33.0 (11.4) (*n* = 30) Placebo: 34.4 (10.6) (*n* = 31) Mean (SD)	NA	Antibiotics: 26.1 (14.7) (*n* = 30) Placebo: 31.3 (14.3) (*n* = 28) −4.5 (−10.6 to 1.6) (*n* = 62) Mean (SD)	NA	*p* = 0.14		Low
	*A mixed patient cohort with MC1 and MC2*								
	Bråten et al., 2019 (Amoxicillin: *n* = 89) (Placebo; *n* = 91)	RMDQ (Both MC1 and MC2) RMDQ between‐group differences (95% CI) (Both MC1 and MC2)	Antibiotics: 12.8 (4.7) (*n* = 88) Placebo: 12.8 (3.7) (*n* = 90) Mean (SD)	Antibiotics: 10.3 (5.8) (*n* = 85) Placebo: 12.4 (4.4) (*n* = 87) −1.9 (−3.2 to −0.7) (*n* = 180) Mean (SD)	Antibiotics: 9.0 (6.2) (*n* = 85) Placebo: 10.7 (5.6) (*n* = 84) −1.6 (−3.1 to 0.0) (*n* = 180) Mean (SD)	NA	Between‐group immediate post‐treatment: *p* = 0.003 Between‐group at 1‐year FU; *p* = 0.04		Low
	Bråten et al., 2019 (Amoxicillin: *n* = 89) (Placebo; *n* = 91)	ODI (Both MC1 and MC2) ODI between‐group differences (95% CI) (Both MC1 and MC2)	Antibiotics: 31.9 (11.4) (*n* = 88) Placebo: 31.8 (10.3) (*n* = 89) Mean (SD)	Antibiotics: 26.6 (14.7) (*n* = 86) Placebo: 30.4 (10.7) (*n* = 85) −3.8 (−6.7 to −0.9) (*n* = 179) (*p* = 0.0.01) Mean (SD)	Antibiotics: 24.4 (15.0) (*n* = 85) Placebo: 28.9 (14.0) (*n* = 85) −4.8 (−8.3 to −1.4) (*n* = 179) *p* = 0.007 Mean (SD)	NA	Between‐group immediate post‐treatment: *p* = 0.01 Between‐group 1‐year *p* = 0.007		Low
*Clinically significant changes in LBP*‐*related disability*									
LBP‐related disability	*Between*‐*group comparisons*								
	Bråten et al., 2019 (Amoxicillin: *n* = 89) (Placebo; *n* = 91)	Clinically significant decreased in RMDQ (>30% improvement) (Both MC1 and MC2)	NA	NA	Antibiotics 40/84 (48%) Placebo: 24/83 (29%)	NA	*p* = 0.01		Low
	Bråten et al., 2019 (Amoxicillin: *n* = 89) (Placebo; *n* = 91)	Clinically significant decreased in RMDQ (>50% improvement) (Both MC1 and MC2)	NA	NA	At 11‐month FU: Antibiotics 23/84 (27%) Placebo: 18/83 (22%)	NA	*p* = 0.39		Low
	Bråten et al., 2019 (Amoxicillin: *n* = 89) (Placebo; *n* = 91)	Clinically significant decreased in RMDQ (>75% improvement) (Both MC1 and MC2)	NA	NA	At 11‐month FU: Antibiotics 15/84 (19%) Placebo: 7/83 (8%)	NA	*p* = 0.07		Low
*Concomitant clinically significant changes in LBP and LBP*‐*related disability*									
LBP‐related disability and LBP	Al‐Falahi et al., 2014 (Amoxicillin‐clavulanate: *n* = 42) (Placebo; *n* = 29)	Clinically significant decreases in RMDQ (>30% improvement) and LBP (>2 score decreases in LBP rating scale) (MC1 only)	NA	Antibiotics (10 out of 26) Placebo: (0 out of 17)	NA	NA	Not reported		Very low
		12% to 30% decreases in RMDQ and 1 to 2 score decrease in LBP rating scale (MC1 only)	NA	Antibiotics (16 out of 26) Placebo: (0 out of 17)	NA	NA	Not reported		
		6%–13% decrease in RMDQ scores from baseline (MC1 only)	NA	Antibiotics (0 out of 26) Placebo: (4 out of 17)	MA	MA	Not reported		
		No improvement in RMDQ scores or LBP rating scale (MC1 only)	NA	Antibiotics (0 out of 26) Placebo: (8 out of 17)	NA	NA	Not reported		
		6% increases in RMDQ scores from baseline (MC1 only)	NA	Antibiotics (0 out of 26) Placebo: (3 out of 17)	NA	NA	Not reported		
*Global perceived health*									
Global perceived health^a^	*Between*‐*group comparisons*								
	Albert et al., 2013 (Amoxicillin‐clavulanate: *n* = 90) (Placebo: *n* = 72)	7‐point global perceived effect scale (improvement) (MC1 only)	NA	NA	Antibiotics: 39 ± 38.4% (*n* = 77) Placebo: 1.8 ± 31.7% (*n* = 67)	NA	*p* = 0.0001		Low
	Bråten et al., 2019 (Amoxicillin: *n* = 89) (Placebo; *n* = 91) (Both MC1 and MC2)	Improved (Both MCI and MC2)	NA	NA	Antibiotics: 24/85 (28%) Placebo: 18/84 (21%)	NA	*p* = 0.39		Low
		Unchanged (Both MCI and MC2)	NA	NA	Antibiotics: 58/85 (68%) Placebo: 60/84 (71%)	NA			
		Worsen (Both MCI and MC2)	NA	NA	Antibiotics: 3/85 (4%) Placebo: 4/84 (7%)	NA			
Patient satisfaction at 1 year	Bråren et al., 2019 (Amoxicillin: *n* = 89) (Placebo; *n* = 91) (Both MC1 and MC2)	Satisfied (Both MCI and MC2)	NA	NA	Antibiotics: 35/85 (41%) Placebo: 18/84 (21%)	NA	*p* = 0.52		Low
		Somewhat satisfied (Both MCI and MC2)	NA	NA	Antibiotics: 8/85 (9%) Placebo: 9/84 (11%)	NA			
		Neither satisfied nor dissatisfied (Both MCI and MC2)	NA	NA	Antibiotics: 34/85 (40%) Placebo: 35/84 (42%)	NA			
		Somewhat dissatisfied (Both MCI and MC2)	NA	NA	Antibiotics: 5/85 (6%) Placebo: 4/84 (5%)	NA			
		Dissatisfied (Both MCI and MC2)	NA	NA	Antibiotics: 3/85 (4%) Placebo: 8/84 (10%)	NA			
Bothersomeness by LBP	Albert et al., 2013 (Amoxicillin‐clavulanate: *n* = 90) (Placebo: *n* = 72)	Bothersomeness scale (0–10) (MC1 only)	Antibiotics: 7 (6 and 8) (*n* = 90) Placebo: 8 (5 and 9) (*n* = 72) Median (25th and 75th percentile)	NA	Antibiotics: 3 (2 and 5) (*n* = 77) Placebo: 6 (4 and 8) (*n* = 67) Median (25th and 75th percentile)	NA	*p* = 0.0014		Low
*Quality of life*									
Quality of life	*Between*‐*group comparisons*								
	Patients with MC1 only								
	Albert et al., 2013 (Amoxicillin‐clavulanate: *n* = 90) (Placebo: *n* = 72)	EQ‐5D thermometer (1–100, 100 is the best) (MC1 only)	Antibiotics: 59 (40;70) (*n* = 90) Placebo: 60 (40;75) (*n* = 72) Median (25th and 75th percentile)	Antibiotics: 65 (40;79) (*n* = 76) Placebo: 60 (40;75) (*n* = 67) Median (25th and 75th percentile)	Antibiotics: 75 (54;90) (*n* = 77) Placebo: 60 (39;74) (*n* = 67) Median (25th and 75th percentile)	NA	*p* = 0.0014		Low
	Bråten et al., 2019 (Amoxicillin: *n* = 58) (Placebo; *n* = 60)	EQ5D‐5L (MC1 only) EQ‐5D between‐group differences (95% CI) (MC1 only)	Antibiotics: 0.55 (0.18) (*n* = 58) Placebo: 0.56 (0.16) (*n* = 60) Mean (SD)	NA	Antibiotics: 0.66 (0.21) (*n* = 55) Placebo: 0.60 (0.21) (*n* = 55) 0.07 (0.01 to 0.14) (*n* = 118) Mean (SD)	NA	*p* = 0.03		Low
	Bråten et al., 2019 (Amoxicillin: *n* = 31) (Placebo; *n* = 31)	EQ5D‐5L (MC2 only) EQ‐5D between‐group differences (95% CI) (MC2 only)	Antibiotics: 0.53 (0.21) (*n* = 31) Placebo: 0.51 (0.20) (*n* = 31) Mean (SD)	NA	Antibiotics: 0.61 (0.24) (*n* = 29) Placebo: 0.55 (0.25) (*n* = 28) 0.06 (−0.04 to 0.16) (*n* = 62) Mean (SD)	NA	*p* = 0.22		Low
	Bråten et al., 2019 (Amoxicillin: *n* = 89) (Placebo; *n* = 91)	EQ5D‐5L (Both MC1 and MC2) EQ5D‐5L between‐group differences (95% CI) (Both MC1 and MC2)	Antibiotics: 0.55 (0.19) (*n* = 89) Placebo: 0.54 (0.18) (*n* = 91) Mean (SD)	Antibiotics: 0.60 (0.22) (*n* = 85) Placebo: 0.54 (0.21) (*n* = 83) 0.06 (0.00 to 0.11) (*n* = 180 Mean (SD)	Antibiotics: 0.65 (0.22) (*n* = 84) Placebo: 0.58 (0.22) (*n* = 83) 0.07 (0.02–0.12) (*n* = 180) Mean (SD)	NA	Between‐group immediate posttreatment *p* = 0.04 Between‐group at 1‐year FU: *p* = 0.01		Low
*Medications*									
Concomitant drug use	Between‐group comparisons								
	Bråten et al., 2019 (Amoxicillin: *n* = 89) (Placebo; *n* = 91)	Analgesics (Both MC1 and MC2)	Antibiotics: 62/89 (70%) Placebo: 61/91 (67%)	NA	Antibiotics: 61/89 (69%) Placebo: 67/91 (74%)	NA	*p* = 0.45		Low
		NSAID (Both MC1 and MC2)	Antibiotics: 38/89 (43%) Placebo: 36/91 (40%)	NA	Antibiotics: 38/89 (44%) Placebo: 40/91 (44%)	NA	*p* = 0.99		Low
		Opioids (Both MC1 and MC2)	Antibiotics: 28/89 (31%) Placebo: 27/91 (30%)	NA	Antibiotics: 28/89 (31%) Placebo: 35/91 (38%)	NA	*p* = 0.33		Low
Compliance of consuming 95%–100% of all tablets	Albert et al., 2013 (Amoxicillin‐clavulanate: *n* = 90) (Placebo: *n* = 72)	Daily medication diary (MC1 only)	NA	NA	Antibiotics: 94.8% (*n* = 77) Placebo: 94.0% (*n* = 67)	NA	NS		Low
*Medical consultations and sick leaves*									
Number of days of sick leave last year	Albert et al., 2013 (Amoxicillin‐clavulanate: *n* = 90) (Placebo: *n* = 72)	Counts in the last 12 months (MC1 only)	Antibiotics: 51.0 ± 92 (*n* = 90) Placebo: 42.0 ± 80 (*n* = 72)	NA	Antibiotics: 18.9 ± 61 (*n* = 77) Placebo: 45.4 ± 90 (*n* = 67)	NA	*p* = 0.064		Low
Consulted a doctor because of back pain during the follow‐up year	Albert et al., 2013 (Amoxicillin‐clavulanate: *n* = 90) (Placebo: *n* = 72)	Counts (MC1 only)	NA	NA	Antibiotics: 23.4% (*n* = 90) Placebo: 41.8% (*n* = 72)	NA	*p* = 0.002		Low
Number of medical consultations for LBP	Al‐Falahi et al., 2014 (Amoxicillin‐clavulanate: *n* = 42) (Placebo; *n* = 29)	Counts (MC1 only)	NA	Antibiotics: 7 out of 26 (26%) Placebo: 9 out of 17 (53%)	NA	NA	*p* = 0.0844		Very low
*Adverse events*									
Adverse events	Albert et al., 2013 (Amoxicillin‐clavulanate: *n* = 90) (Placebo: *n* = 72)	Low‐grade gastroenterological complaints (e.g., loose bowel movements, more flatus or burping); middle‐grade events (e.g., loose bowel movements for >3 weeks), severe vomiting causing blood in the vomit.	NA	NA	Antibiotics: 65% Placebo: 23%	NA	Not reported		Low
	Al‐Falahi et al., 2014 (Amoxicillin‐clavulanate: *n* = 42) (Placebo; *n* = 29)	Low‐grade gastroenterological complaints such as loose bowel movements, increased flatus, or infrequent attacks of abdominal colic.	NA	Antibiotics: 60% Placebo: 6%	NA	NA	Not reported		Very low
	Bråten et al., 2019 (Amoxicillin: *n* = 89) (Placebo; *n* = 91)	Count (Both MC1 and MC2)	NA	NA	Antibiotics: 50/89 (56%) Placebo: 31/91 (34%)	NA	*p* = 0.0029		Low
		Severe adverse events	NA	NA	Antibiotics (*n* = 6; 7%) Placebo (*n* = 2; 2%)	NA	Not reported		Low
		Odds ratio (OR) of having adverse events (any grade) with reference to the placebo group	NA	NA	OR = 2.4 (95% CI: 1.02–5.6)	NA	*p* = 0.045		Low
*Lumbar magnetic resonance imaging results*									
Modic 1 change	Albert et al., 2013 (Amoxicillin‐clavulanate: *n* = 90) (Placebo: *n* = 72)	Observed volume 1, minute size (MC1 only)	Antibiotics: 16 Placebo: 31	NA	Antibiotics: 29 Placebo: 24	NA	*p* = 0.05		Low
		Observed volumes 2–4, moderate/large size (MC1 only)	Antibiotics: 126 Placebo: 99	NA	Antibiotics: 113 Placebo: 96	NA	*p* = 0.07		Low
*Blood test results*									
Hemoglobin	Albert et al., 2013 (Amoxicillin‐clavulanate: *n* = 90) (Placebo: *n* = 72)	Serum analysis (mmol/L) value [no. of people exceeding reference values (8.0–11.0 mmol/L)] (MC1 only)	Antibiotics: 8.8 [0] Placebo: 8.7 [0]	NA	Antibiotics: 8.7 [0] Placebo: 8.9 [0]	NA	*p* > 0.05		Low
Erythrocytes	Albert et al., 2013 (Amoxicillin‐clavulanate: *n* = 90) (Placebo: *n* = 72)	Serum analysis value [no. of people exceeding reference values (3.6–5.1)] (MC1 only)	Antibiotics: 4.6 [6] Placebo: 4.6 [11]	NA	Antibiotics: 4.5 [5] Placebo: 4.6 [9]	NA	*p* > 0.05		Low
Erythrocytes vol.fr	Albert et al., 2013 (Amoxicillin‐clavulanate: *n* = 90) (Placebo: *n* = 72)	Serum analysis value [no. of people exceeding reference values (0.34–0.44)] (MC1 only)	Antibiotics: 0.42 [12] Placebo: 0.42 [19]	NA	Antibiotics: 0.42 [12] Placebo: 0.42 [13]	NA	*p* > 0.05		Low
Erythrocytes; vol	Albert et al., 2013 (Amoxicillin‐clavulanate: *n* = 90) (Placebo: *n* = 72)	Serum analysis value [no. of people exceeding reference values (80–100)] (MC1 only)	Antibiotics: 91 [3] Placebo: 92 [2]	NA	Antibiotics: 87 [2] Placebo: 91 [2]	NA	*p* > 0.05		Low
Erythrocytes (B)–Hemoglobin‐(Fe)	Albert et al., 2013 (Amoxicillin‐clavulanate: *n* = 90) (Placebo: *n* = 72)	Serum analysis value (fmol) [no. of people exceeding reference values (1.7–2.2 fmol)] (MC1 only)	Antibiotics: 1.9 [10] Placebo: 2.0 [9]	NA	Antibiotics: 1.9 [1] Placebo: 1.9 [3]	NA	*p* > 0.05		Low
Leucocytes	Albert et al., 2013 (Amoxicillin‐clavulanate: *n* = 90) (Placebo: *n* = 72)	Serum analyses value (/l) (no. of people exceeding reference values (3.0–10.0 × 10^3^/L) (MC1 only)	Antibiotics: 7.0 [3] Placebo: 7.3 [9]	NA	Antibiotics: 7.4 [25] Placebo: 7.4 [8]	NA	*p* > 0.05		Low
Neutrophils	Albert et al., 2013 (Amoxicillin‐clavulanate: *n* = 90) (Placebo: *n* = 72)	Serum analysis value (no. of people exceeding reference values (1.5–7.5 × 10^9^/L) (MC1 only)	Antibiotics: 4.2 [1] Placebo: 4.4 [4]	NA	Antibiotics: 4.5 [4] Placebo: 4.3 [3]	NA	*p* > 0.05		Low
Eosinophils	Albert et al., 2013 (Amoxicillin‐clavulanate: *n* = 90) (placebo: *n* = 72)	Serum analysis value (no. of people exceeding reference values (0.04–0.5 × 10^9^/L) (MC1 only)	Antibiotics: 0.15 [1] Placebo: 0.16 [1]	NA	Antibiotics: 0.2 [4] Placebo: 0.2 [5]	NA	*p* > 0.05		Low
Basophils	Albert et al., 2013 (Amoxicillin‐clavulanate: *n* = 90) (Placebo: *n* = 72)	Serum analysis value (no. of people exceeding reference values (<0.2 × 10^9^/L) (MC1 only)	Antibiotics: 0.04 [0] Placebo: 0.03 [0]	NA	Antibiotics: 0.07 [2] Placebo: 0.04 [0]	NA	*p* > 0.05		Low
Lymphocytes	Albert et al., 2013 (Amoxicillin‐clavulanate: *n* = 90) (Placebo: *n* = 72)	Serum analysis value (no. of people exceeding reference values (1.0–3.5 × 10^9^/L) (MC1 only)	Antibiotics: 2.1 [2] Placebo: 2.1 [2]	NA	Antibiotics: 2.1 [4] Placebo: 2.2 [2]	NA	*p* > 0.05		Low
Monocytes	Albert et al., 2013 (Amoxicillin‐clavulanate: *n* = 90) (Placebo: *n* = 72)	Serum analysis value (no. of people exceeding reference values (0.2–0.8 × 10^9^/L) (MC1 only)	Antibiotics: 0.5 [2] Placebo: 0.5 [4]	NA	Antibiotics: 0.6 [5] Placebo: 0.6 [8]	NA	*p* > 0.05		Low
P/S Creatinine	Albert et al., 2013 (Amoxicillin‐clavulanate: *n* = 90) (Placebo: *n* = 72)	Serum analysis value (no. of people exceeding reference values (62–134 μmol/L) (MC1 only)	Antibiotics: 84 [0] Placebo: 81 [0]	NA	Antibiotics: 82 [0] Placebo: 83 [0]	NA	*p* > 0.05		Low
	Albert et al., 2013 Amoxicillin‐clavulanate (unclear distribution; *n* = 16)	Average serum analysis value [no. of people exceeding reference values (105–205 U/L)] (MC1 only)	Antibiotic: 152 [*n* = 2] Placebo: 155 [*n* = 0]	NA	Antibiotic: 153 [*n* = 3] Placebo: 152 [*n* = 2]	NA	*p* > 0.05		Low
Alkaline phosphatase, original method	Albert et al., 2013 (unclear distribution; *n* = 13) Amoxicillin‐clavulanate	Serum analysis values (no. of people exceeding reference values (80–275 U/L) (MC1 only)	Antibiotic: 65 [0] Placebo: 64 [0]	NA	Antibiotic: 74 [1] Placebo: 63 [0]	NA	NS		Low

^a^ The calculated percentages reported in the article were wrong based on the number of reported participants.

Regarding leg pain, low‐quality evidence supported that Amoxicillin‐clavulanate was significantly better than placebo in improving leg pain in CLBP patients with MC1, or both MC1 and MC2 at the 12‐month follow‐up. However, only very low‐quality evidence supported that patients with MC1 had clinically significantly better LBP reduction than placebo immediately post‐treatment (Table 3, Table A2).

### 3.3 Between‐group comparisons in improving disability among patients with CLBP

**FIGURE 4:**
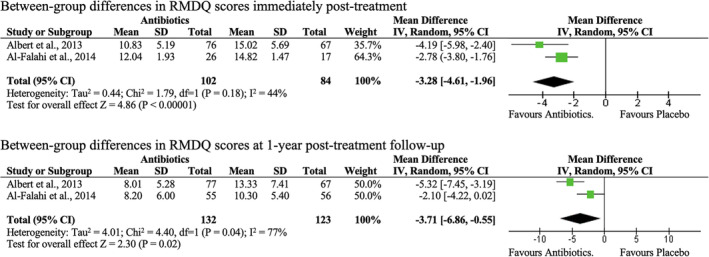
Forest plot of effects of antibiotics versus placebo on Roland Morris Disability Questionnaire (RMDQ) scores of patients with chronic low back pain and type 1 Modic changes immediately post‐treatment, and at the 12‐month follow‐up.

Low‐quality evidence from a meta‐analysis suggested that 100 days of oral antibiotics were significantly better than placebo in improving RMDQ scores of CLBP patients with MC1 (MD: −3.20; 95% CI: −4.61 to −1.96; *I* ^2^ = 44%) immediately post‐treatment (Figure 4). Likewise, there was moderate evidence that 90 or 100 days of oral antibiotic was significantly better than placebo in lowering RMDQ scores in CLBP patients with MC1 at the 12‐month follow‐up (MD: −0.71; 95% CI: −6.86 to −0.55; *I* ^2^ = 77%). Low‐quality evidence from one included RCT also showed that patients with CLBP and MC1 taking antibiotics had significantly better improvements in ODI scores than placebo controls at the 12‐month follow‐up (Table 3, Table A2). Similar post‐treatment findings were observed in a mixed cohort of patients with MC1 and MC2. Interestingly, one included RCT found that compared to the placebo group, a significantly higher percentage of a patient cohort with MC1 and MC2 in the intervention group displayed clinically significant improvement in disability (>30% reduction in baseline RMDQ scores) at the 12‐month follow‐up.^29^

### 3.4 Between‐group comparisons of bothersome by LBP and global perceived health

Low‐quality evidence suggested that CLBP patients with MCI in the antibiotic group experienced significantly less LBP‐related bothersome and significantly better perceived general improvement than placebo controls at the 12‐month follow‐up (Table 3, Table A2). Low‐quality evidence supported no significant between‐group difference in global post‐treatment improvement in a mixed cohort of CLBP patients with MC1 and MC2.

### 3.5 Between‐group comparisons in posttreatment health‐related quality of life and patient satisfaction

Low‐quality evidence supported that patients with MC1 or a mixed cohort of patients with MC1 and MC2 in the intervention group displayed significantly better quality of life (as measured by EQ5D‐5L or EQ‐5D thermometer) than placebo controls at the 12‐month follow‐up. However, low‐quality evidence suggested no such significant between‐group difference in patients with MC2 (Table A2).

Additionally, low‐quality evidence from one included RCT substantiated that patients with MC1 or a mixed cohort of patients with MC1 and MC2 in the antibiotic group showed significantly better but small improvement in the quality‐adjusted life‐year (QALY) than their placebo controls at the 12‐month follow‐up. However, the same study found no significant between‐group difference in patient satisfaction in a mixed cohort of patients with MC1 and MC2 at the 12‐month follow‐up (Table A2).

### 3.6 Between‐group comparisons of post‐treatment changes in MC1 size or serum content

Low‐quality evidence suggested that there was no significant between‐group difference in the size of MC1 volume or serum analysis results at the 12‐month follow‐up (Table 3, Table A2).

### 3.7 Between‐group comparisons of drug use, sick leave, or medical consultations

Low‐quality evidence from one RCT supported no significant between‐group difference in the usage of analgesics, non‐steroid anti‐inflammatory drugs, or opioids among patients with MC1 or MC2 over the 12‐month study period. Likewise, there was low‐quality evidence that the Amoxicillin‐clavulanate and placebo groups had a similar number of sick leave days over 1 year. Conversely, low‐quality evidence supported that patients with MC1 in the intervention group had significantly less LBP‐related medical consultation than those in the placebo group over 1 year (Table A2).^28^

### 3.8 Between‐group comparisons of adverse events

There was low‐quality evidence that oral antibiotics caused significantly more adverse events (low‐grade gastroenterological complaints) than placebo pills.

### 3.9 Within‐group improvements in pain, disability, global perceived health, and quality of life following oral antibiotic treatments

Very low‐quality evidence from case series showed that oral antibiotics yielded both clinically and statistically significant decreases in LBP among patients with MC1, MC2, or mixed MCs immediately post‐treatment, and at the 12‐ or 24‐month follow‐ups (Table 4, Table A3). Very low‐quality evidence also supported that Amoxicillin‐clavulanate significantly reduced the number of days with LBP or the number of LBP‐related sleep disturbances. Likewise, there was very low‐quality evidence that Amoxicillin‐clavulanate significantly reduced leg pain in a mixed cohort of patients with MC1 and MC2 immediately post‐treatment, and at 12‐ and 24‐month follow‐ups, although these changes were not clinically significant.

**TABLE 4:** The effects of antibiotics on clinical outcomes among people with chronic low back pain in before‐ and after‐studies.

	Study (initial sample size), study design	Measurement tools	Baseline	End of treatment	1‐year follow‐up	2‐year follow‐up	*p*‐value	Effect (Pooled)	Strength of evidence
*Within*‐*group changes in low back pain immediate post*‐*treatment*									
LBP	*Patients with MC1 only*								
	Albert et al., 2008 (*n* = 29), uncontrolled Amoxicillin‐clavulanate	LBP rating scale [current + (worst + mean in the last 2 weeks)] (0 is the best; 30 is the worst) (MC1 only)	9 (6 and 15) Median (25th and 75th percentile)	5 (1.5 and 9.5) Median (25th and 75th percentile)	NA	NA	*p* < 0.001		Very low
	Palazzo et al., 2017 (*n* = 28) Uncontrolled Amoxicillin‐clavulanate	NPRS (MC1 only)	6.1 (range: 2–9)	5.0 (range: 0–9)	NA	NA	*p* = 0.048		
	*Patients with MC1*, *MC2*, *and/or mixed MCs*								
	Albert et al., 2017 (*n* = 1024) Uncontrolled Amoxicillin‐clavulanate	LBP rating scale [current + (worst + mean in the last 2 weeks)/3] (0 is the best) (MC1, MC2, or mixed MC)	6.0 (1.8) Mean (SD)	4.0 (2.1) (*n* = 987) Mean (SD)	NA	NA	*p* < 0.05		Very low
*Within*‐*group changes at the 11*‐ *or 12*‐*month follow*‐*up*									
LBP	Patients with MC1 only								
	Albert et al., 2008 (*n* = 29), uncontrolled Amoxicillin‐clavulanate	LBP rating scale [current + (worst + mean in the last 2 weeks)] (0 is the best; 30 is the worst) (MC1 only)	9 (6 and 15) Median (25th and 75th percentile)	NA	At 11‐month FU: 5 (2.5 and 12) Median (25th and 75th percentile)	NA	*p* < 0.001		Very low
	Albert et al., 2017 (*n* = 1024) Uncontrolled Amoxicillin‐clavulanate	LBP rating scale [current + (worst + mean in the last 2 weeks)/3] (0 is the best) (MC1, MC2, or mixed MC)	6.0 (1.8) Mean (SD)	NA	3.3 (2,3) (*n* = 902) Mean (SD)	NA	*p* < 0.05		
*Within*‐*group pain changes at the 24*‐*month follow*‐*up for patients with MC1*, *MC2*, *or mixed MCs*)									
LBP	Albert et al., 2017 (*n* = 1024) Uncontrolled Amoxicillin‐clavulanate	LBP rating scale [current + (worst + mean in the last 2 weeks)/3] (0 is the best) (MC1, MC2, or mixed MC)	6.0 (1.8) Mean (SD)	NA	NA	3.0 (2.3) (*n* = 270) Mean (SD)	*p* < 0.05		Very low
*Within*‐*group changes in pain duration*, *sleep*, *or morning stiffness in patients with MC1*									
Number of days with LBP in the last 100 days	Albert et al., 2008 (*n* = 27), uncontrolled Amoxicillin‐clavulanate	Counts in the last 100 days (MC1 only)	100 (25 and 100) Median (25th and 75th percentile)	35 (7 and 35) Median (25th and 75th percentile)	At 11‐month FU: 20 (10 and 84) Median (25th and 75th percentile)	NA	*p* < 0.001 immediately after treatment and at the 11‐month FU		Very low
No. of time per night awakened by LBP	Palazzo et al., 2017 (*n* = 28) Uncontrolled Amoxicillin‐clavulanate	Number of night‐time awakening (MC1 only)	1.0 (range: 0–3)	0.5 (range: 0–1)	NA	NA	*p* = 0.020		Very low
Morning stiffness duration	Palazzo et al., 2017 (*n* = 28) Uncontrolled Amoxicillin‐clavulanate	Estimated time	32.0 (range: 0–120) min	34.0 (range: 0–240) min	NA	NA	*p* = 0.937		Very low
**Leg pain**									
*Within*‐*group changes immediate post*‐*treatment*									
Leg pain	Albert et al., 2017 (*n* = 1024) Uncontrolled Amoxicillin‐clavulanate	NPRS (MC1, MC2, mixed MC)	3.7 (2.7) (*n* = 1024) Mean (SD)	2.3 (2.4) (*n* = 987) Mean (SD)	NA	NA	*p* < 0.05		Very low
*Within*‐*group changes at the 12*‐*month or 24*‐*month follow*‐*up*									
Leg pain	Albert et al., 2017 (*n* = 1024) Uncontrolled Amoxicillin‐clavulanate	NPRS (MC1, MC2, mixed MC)	3.7 (2.7) (*n* = 1024) Mean (SD)	NA	2.0 (2.4) (*n* = 602) Mean (SD)	NA	*p* < 0.05		Very low
	Albert et al., 2017 (*n* = 1024) Uncontrolled Amoxicillin‐clavulanate	NPRS (MC1, MC2, mixed MC)	3.7 (2.7) (*n* = 1024) Mean (SD)	NA	NA	1.9 (2.3) (*n* = 270)	*p* < 0.05		Very low
*LBP*‐*related disability*									
LBP‐related disability	*Within*‐*group changes in patients with MC1 only*								
	Albert et al., 2008 (*n* = 29), uncontrolled Amoxicillin‐clavulanate	RMDQ (0 is the best) (MC1 only)	8 (4.5 and 13.0) Median (25th and 75th percentile: 5)	4 (0.5 and 9.0) Median (25th and 75th percentile:)	At 11‐month FU: 5 Median (25th and 75th percentile: 1–10)	NA	Immediate posttreatment *p* < 0.001 1‐year FU, *p* < 0.001		Very low
	Gupta et al., 2017 (Amoxicillin‐clavulanate: *n* = 9)	ODI (MC1 only)	38.0 (32.0 and 48.0) Median (25th and 75th percentile)	29.0 (26.0 and 48.0) Median (25th and 75th percentile)	25.0 (22.0 and 46.0) Median (25th and 75th percentile)	NA	Immediate: posttreatment *p* = 0.484 1‐year FU; *p* = 0.135		Very low
	Albert et al., 2008 (*n* = 28), uncontrolled Amoxicillin‐clavulanate	Patient‐specific function scale (30 is the best) (MC1 only)	14 (10.5 and 18.5) Median (25th and 75th percentile)	21 (17.5 and 24.5) Median (25th and 75th percentile:)	At 11‐month FU: 21 (13.0 and 25.0) Median (25th and 75th percentile)	NA	Immediate posttreatment *p* < 0.001 1‐year FU, *p* < 0.001		Very low
	*Within*‐*group changes in patients with MC1*, *MC2*, *and mixed MCs*								
	Albert et al., 2017 (*n* = 1024) Uncontrolled Amoxicillin‐clavulanate	RMDQ (MC1, MC2, mixed MC)	15.5 (4.4) (*n* = 1024) Mean (SD)	11.7 (7.5) (*n* = 987) Mean (SD)	8.3 (6.2) (*n* = 602) Mean (SD)	7.4 (7.1) (*n* = 270) Mean (SD)	*p* < 0.05 at all time points		Very low
*Clinically significant changes in LBP*‐*related disability*									
LBP‐related disability	*Within*‐*group comparisons*								
	Albert et al., 2008 (*n* = 29), uncontrolled Amoxicillin‐clavulanate	Clinically improved (RMDQ ≥30% improvement) Unchanged Clinically worsen (> 30% worsening) (MC1 only)	NA	*N* = 18 (62%) *N* = 10 (34.5%) *N* = 1 (3.5%)	At 11‐month FU: 18 (62%) 9 (31.0%) 2 (7.0%)	NA	Not reported		Very low
*Global perceived health*									
Global perceived health	*Within*‐*group comparisons*								
	Albert et al., 2008 (n = 29), uncontrolled Amoxicillin‐clavulanate	Much better or cured Moderately better Unchanged (MC1 only)	NA	*N* = 15/29 (52%) *N* = 7/29 (24%) *N* = 7/29 (24%)	NA	NA	*p* < 0.05		Very low
	Palazzo et al., 2017 (*n* = 28)^a^ Uncontrolled Amoxicillin‐clavulanate	Improvement ≥70% Improvement ≥50% No improvement	NA	4/28 (14.3%) 6/28 (21.4%) 16/28 (57.1%)	4/27 (14.5%) 6/27 (22.2%) 19/27 (70.4%)	NA	Not reported		Very low
*Quality of life*									
Quality of life	*Within*‐*group comparisons*								
	Albert et al., 2017 (*n* = 1024) Uncontrolled Amoxicillin‐clavulanate	EQ‐5D thermometer (0–100) (MC1, MC2, mixed MC)	48.1 (20.1) Mean (SD)	59.2 (20.7) Mean (SD)	71.8 (40.8) Mean (SD)	70.3 (38.3) Mean (SD)	*p* values not reported		Very low
*Medications*									
Concomitant drug use	*Within*‐*group comparisons*								
	Palazzo et al., 2017 (*n* = 28) uncontrolled Amoxicillin‐clavulanate	Analgesics (MC1 only)	25/28 (89.2%)	19/28 (70.4%)	NA	NA	*p* = 0.751		Very low
	Albert et al., 2017 (*n* = 1024) Uncontrolled Amoxicillin‐clavulanate	Analgesics usage in the last week (MC1, MC2, mixed MC)	23.2 (28.7) Mean (SD)	13.2 (23.1) Mean (SD)	13,2 (23.1) Mean (SD)	6.8 (19.7) Mean (SD)	*p* values were not reported		Very low
*Within*‐*group changes in MC1*									
Modic 1 change	Gupta et al., 2017 Amoxicillin‐clavulanate (*n* = 8)	Endplate volume (MC1 only)	NA	NA	Improved (*n* = 2) No change (*n* = 2) Increased MC1 (*n* = 4)	NA	Not reported		Very low
*Within*‐*group changes in blood test results*									
Hemoglobin	Albert et al., 2008 (*n* = 29), uncontrolled Amoxicillin‐clavulanate	Serum analyses (no. of people exceeding reference values (8.0–11.0 mmol/L) (MC1 only)	0	2	NA	NA	*p* > 0.05		Very low
Leucocytes	Albert et al., 2008 (*n* = 29), uncontrolled Amoxicillin‐clavulanate	Serum analyses (no. of people exceeding reference values (3.0–10.0 × 10^9^/L), (MC1 only)	1	3	NA	NA	*p* > 0.05		Very low
Neutrophils	Albert et al., 2008 (*n* = 29), uncontrolled Amoxicillin‐clavulanate	Serum analyses (no. of people exceeding reference values (1.5–7.5 × 10^9^/L) (MC1 only)	0	1	NA	NA	*p* > 0.05		Very low
Eosinophils	Albert et al., 2008 (*n* = 29), uncontrolled Amoxicillin‐clavulanate	Serum analyses (no. of people exceeding reference values (0.04–0.5 × 10^9^/L) (MC1 only)	0	1	NA	NA	*p* > 0.05		Very low
Basophils	Albert et al., 2008 (*n* = 29), uncontrolled Amoxicillin‐clavulanate	Serum analyses (no. of people exceeding reference values (<0.2 × 10^9^/L) (MC1 only)	0	1	NA	NA	*p* > 0.05		Very low
Lymphocytes	Albert et al., 2008 (*n* = 29), uncontrolled Amoxicillin‐clavulanate	Serum analyses (no. of people exceeding reference values (1.0–3.5 × 10^9^/L) (MC1 only)	0	2	NA	NA	*p* > 0.05		Very low
Monocytes	Albert et al., 2008 (*n* = 29), uncontrolled Amoxicillin‐clavulanate	Serum analyses (no. of people exceeding reference values (0.2–0.8 × 10^9^/L) (MC1 only)	3	1	NA	NA	*p* > 0.05		Very low
P/S Creatinine	Albert et al., 2008 (*n* = 29), uncontrolled Amoxicillin‐clavulanate	Serum analyses (no. of people exceeding reference values (62–134 μmol/L) (MC1 only)	0	0	NA	NA	*p* > 0.05		Very low
Lactate dehydrogenase, original method	Albert et al., 2008 (*n* = 13), uncontrolled Amoxicillin‐clavulanate	Serum analyses (no. of people exceeding reference values (150–500 U/L) (MC1 only)	0	0	NA	NA	*p* > 0.05		Very low
Lactate dehydrogenase, new method	Albert et al., 2008 (*n* = 16), uncontrolled Amoxicillin‐clavulanate	Serum analyses (no. of people exceeding reference values (105–205 U/L) (MC1 only)	7	8	NA	NA	*p* > 0.05		Very low
Alkaline phosphatase, original method	Albert et al., 2008 (*n* = 13), uncontrolled Amoxicillin‐clavulanate	Serum analyses (no. of people exceeding reference values (80–275 U/L) (MC1 only)	0	0	NA	NA	*p* > 0.05		Very low
Alkaline phosphatase, new method	Albert et al., 2008 (*n* = 16), uncontrolled Amoxicillin‐clavulanate	Serum analyses (no. of people exceeding reference values (35–105 U/L) (MC1 only)	3	2	NA	NA	*p* > 0.05		Very low
C‐reactive protein	Albert et al., 2008 (*n* = 29), uncontrolled Amoxicillin‐clavulanate	Serum analyses (no. of people exceeding reference values (<10 mg/L) (MC1 only)	5	3	NA	NA	*p* > 0.05		Very low

^a^ The calculated percentages reported in the article were wrong based on the number of reported participants.

Very low‐quality evidence supported that 100 days of Amoxicillin‐clavulanate treatment significantly improved RMDQ scores or patient‐specific function scale scores in CLBP patients with MC1 immediately post‐treatment and at the 12‐month follow‐up (Table 4, Table A3). Likewise, very low‐quality evidence substantiated that Amoxicillin‐clavulanate significantly improved RMDQ scores immediately after treatment, and at the 12‐ and 24‐month follow‐ups in CLBP patients with MC1, MC2, and mixed MCs. Very low‐quality evidence supported that up to 76% of patients with MC1 reported moderately better, much better, or even cured LBP after 100 days of Amoxicillin‐clavulanate. However, very low‐quality evidence suggested that patients did not show significant changes in white blood cell test results after taking a course of Amoxicillin‐clavulanate (Table 4, Table A3).

### 3.10 Sensitivity analyses

After removing the RCT with high risk of bias,^36^ the quality of evidence regarding the conclusion regarding the effects of Amoxicillin‐clavulanate against placebo in improving LBP intensity and RMDQ in people with CLBP remained unchanged. However, there was no more evidence to support that Amoxicillin‐clavulanate yielded clinically significantly better LBP reduction than placebo. Likewise, the removal of a poor‐quality case series did not alter our conclusion on the within‐group improvements in clinical outcomes following antibiotic treatments.^40^

## 4 DISCUSSION

This is the first systematic review and meta‐analysis to summarize the efficacy, and safety of antibiotic use in improving clinical outcomes in patients with CLBP who exhibit MCs on MRI. Results from three RCTs indicated that patients with MC1 receiving oral antibiotics (i.e., amoxicillin or amoxicillin/clavulanate) experienced significantly better pain reduction immediately post‐treatment than placebo controls. While no significant between‐group differences in LBP intensity were noted at the 12‐month follow‐up, the prevalence of constant back pain, LBP‐related sleep disturbance, positive cranial compression testing, and pain during lumbar flexion/extension among the antibiotic cohort showed significantly better improvements than the placebo group. Compared to placebo, antibiotics yielded significantly better leg pain reduction in patients with MC1, or mixed MC1 and MC2 immediately post‐treatment and at the 12‐month follow‐up. However, only patients with MC1 displayed clinically significant between‐group differences. Similarly, oral antibiotics achieved significantly greater reductions in LBP‐related disability among patients with MC1 immediately and 12 months following treatments. Additionally, a larger proportion of patients with MC1 and MC2 who took antibiotics experienced clinically significant improvement than the placebo group. Compared to patients with MC1 taking placebo pills, those receiving oral antibiotics perceived significantly better quality‐of‐life at the 12‐month follow‐up; however, no such difference was observed among patients with MC2. Despite various observed benefits of antibiotics, their evidence was very low to low. Additionally, antibiotics were not better than placebo in improving MC1 size or inflammatory biomarker profiles at the 12‐month follow‐up. Therefore, the current findings should be interpreted with caution.

Causes of CLBP are multifactorial (demographic, environmental, hormonal, degenerative, biomechanical, and genetic factors).^42^, ^43^, ^44^, ^45^, ^46^, ^47^, ^48^, ^49^ Despite significant research efforts devoted to developing pharmacological, nonpharmacological, surgical, and psychosocial therapies for treating LBP, the prevalence of CLBP, as well as its substantial physical and socioeconomic consequences have continued to increase.^50^, ^51^, ^52^, ^53^, ^54^, ^55^ Although current CLBP treatment guidelines recommend avoiding the use of opioids when possible, both opioid prescription rates and long‐term use patterns remain to be a concern in recent years.^56^, ^57^ The complex etiology of CLBP is further evidenced by the presence of potential low‐grade infection of intervertebral disc, suggesting microbiome‐targeted therapies as new potential treatment avenues.^12^, ^23^, ^28^ Our findings regarding patients receiving antibiotics experiencing greater improvements in pain and LBP‐related disability appear to support this notion. This concept is particularly interesting as it relates to MCs, an underlying inflammation and bone edema that may be mediated by bacterial cytokine and propionic acid production.^27^ While MCs and associated endplate abnormalities are known to play a significant role in CLBP, meriting their own specific code in the updated ICD‐10 coding system to designate “vertebrogenic LBP,” clinical effects of antibiotic treatment for LBP depend on MC types.^17^ Notably, patients with MC1 demonstrated significantly better LBP, leg pain, LBP‐related disability, perceived improvement, and quality of life following amoxicillin/amoxicillin/clavulanate treatment than those with MC2 and mixed‐type MCs, signifying the progression of fatty replacement of the vertebral red bone marrow may reduce patients' responsiveness to antibiotic therapy. Accordingly, accurate identification and classification of MCs may help clinicians make informed treatment decisions and provide more personalized and targeted therapies.

While low to moderate‐quality evidence supports that oral amoxicillin‐clavulanate or amoxicillin are superior to placebo in reducing LBP/LBP‐related disability in patients with CLBP and concomitant MC1, it remains unclear whether the improvements are attributed to the reduced infections or inflammation in the body (e.g., gums, skin, gut, and/or endplates) following 3 months of antibiotics. This uncertainty is supported by the absence of more reduction in MC1 size in the antibiotic group as compared to placebo.^28^, ^58^ Therefore, future RCTs should compare the clinical effectiveness of oral antibiotics with other active interventions (e.g., NSAID) in treating CLBP patients with MC1.

The inherent consequences of oral antibiotics (e.g., resistance, adverse effects, and costs) may hinder its application as a CLBP treatment.^59^, ^60^, ^61^ Past research has illustrated a direct correlation between antibiotic consumption and resistance at a country‐wide level.^61^ Two meta‐analyses reported that greater antibiotic consumption was associated with more antibiotic resistance [odds ratios (ORs) ranging from 2.3 to 2.5], although this resistance appeared to diminish within 12 months (OR 1.33, 95% CI 1.2–1.5).^59^, ^60^ Similar research also suggests a positive association between dosages of antibiotics and adverse effects, which concurred with our analyses.^62^ That said, antibiotics (including amoxicillin) are often used for 3–4 months before the re‐evaluation of the acne treatment.^63^ These durations exceed 100 days of antibiotic treatment for CLBP. However, prior research showed that oral amoxicillin was not more cost‐effective than placebo in treating CLBP, further high‐quality RCTs are warranted to compare the cost‐effectiveness between antibiotics and other active CLBP treatments in different healthcare settings.

### 4.1 Strengths and limitations

The strengths of the present review lie in its comprehensive literature search, pre‐registered research protocol, as well as summary and analyses regarding the role of antibiotics in treating CLBP. Specifically, the strengths stem from standardized screening and data extraction procedures, the use of validated risk of bias assessment tools, and the GRADE method to synthesize evidence, as well as reporting according to the PRISMA guidelines.

The present review is not without limitations. First, two included studies (one RCT and one case series) had 29% to 41% attrition rates, which could have confounded the results.^36^, ^37^ Second, one included RCT had a high risk of bias that might have affected our results.^36^ However, our sensitivity analysis yielded the same conclusion after discarding its findings. Third, the favorable findings in the included case series might be related to natural history rather than the effects of antibiotics. Fourth, because our review only included two high‐quality small‐scale Danish and Norwegian RCTs, our findings may not be generalized to other patient populations (e.g., older patients) or healthcare settings.^64^ Fifth, although our findings revealed consistent evidence for the benefits of oral amoxicillin/amoxicillin‐clavulanate in treating CLBP patients with MC1, different imaging strengths, or varying MC infiltration may yield differential patients' responses to antibiotics. Sixth, since antibiotics‐related side effects might cause participants to recognize the use of antibiotics, the existing findings should be interpreted with caution.

## 5 CONCLUSIONS

This systematic review and meta‐analysis are the first of such robust study design, to our knowledge, to assess the efficacy and safety of antibiotic use in improving pain, disability, and quality of life in patients with CLBP who exhibit MCs on MRI. Using antibiotics to treat CLBP remains a topic of vehement debate in the literature. Although existing limited evidence suggested that antibiotic treatment might significantly improve back/leg pain, pain‐related disability, and quality of life among patients with MC1, most improvements were only statistically significant and their effects on patients with MC2 or mixed‐type MCs were less prominent. Importantly, the benefits of antibiotic treatment are offset by a higher rate of drug‐related adverse events and increased cost; thus, it remains too early to recommend modification of clinical treatment guidelines to promote their systematic use for treatment of CLBP. Further large‐scale RCTs are warranted to compare the clinical‐ and cost‐effectiveness of oral antibiotics and other active treatments in patients with CLBP with concomitant MC1 on imaging.

## CONFLICT OF INTEREST STATEMENT

The authors have no financial or competing interests concerning this work to disclose.

## APPENDIX 1 SEARCH STRATEGIES

**TABLE A1:** Medline.

1	Low* back pain OR LBP OR back pain OR lumbago OR lumbar spine pain OR Sciatica
2	Lumbar OR lower back OR back OR low back
3	Pain OR agony
4	2 AND 3
5	1 OR 4
6	Chronic* OR persist* OR continu*
7	5 AND 6
8	Disc* OR Disk* OR discogen*
9	Degenerat* OR herniat* OR sequest* OR protru* OR bulg* OR prolap* OR extru*
10	8 AND 9
11	degenerative disc disease* OR DDD
12	10 OR 11
13	Modic chang*
14	vertebral body marrow OR vertebral marrow OR intravertebral edema OR vertebral edema OR intravertebral oedema OR vertebral oedem* OR endplate *chang* OR endplate oedema OR end*‐*plate oedema OR endplate edema OR end*‐*plate edema OR endplate NEAR/3 lesion* OR endplate NEAR/3 change* OR vertebral marrow NEAR/3 lesion*
15	13 OR 14
16	12 OR 15
17	MRI OR magnetic resonan*
18	16 AND 17
19	Pyogen* OR infect* OR inflam* OR discitis OR spondylodiscitis OR Cutibacterium *acne** OR C acne* OR bacteri* OR Propionibacterium OR P* ***acnes*** OR anaerobic bacteri* OR low‐virulent
20	Oral antibiotic* OR amoxicillin‐clavulanate OR amoxicillin OR amoxicillin‐clavulanat* OR Bioclavid
21	7 AND 18 AND 20
22	7 AND 18 AND 19 AND 20
23	7 AND 19 AND 20
24	21 OR 22 OR 23

**TABLE A2:** Embase

No.	Query
#24	#21 OR #22 OR #23
#23	#7 AND #19 AND #20
#22	#7 AND #18 AND #19 AND #20
#21	#7 AND #18 AND #20
#20	oral AND antibiotic* OR ‘amoxicillin clavulanate’ OR ‘amoxicillin’/exp OR amoxicillin OR ‘amoxicillin clavulanat*’ OR ‘bioclavid’/exp OR bioclavid
#19	((((pyogen* OR infect* OR inflam* OR ‘discitis’/exp OR discitis OR ‘spondylodiscitis’/exp OR spondylodiscitis OR ‘cutibacterium’/exp OR cutibacterium) AND acne* OR c) AND acne* OR bacteri* OR ‘propionibacterium’/exp OR propionibacterium OR p*) AND acnes OR anaerobic) AND bacteri* OR ‘low virulent’
#18	#16 AND #17
#17	(‘mri’/exp OR mri OR magnetic) AND resonan*
#16	#12 OR #15
#15	#13 OR #14
#14	(((((((((((vertebral AND (‘body’/exp OR body) AND (‘marrow’/exp OR marrow) OR vertebral) AND (‘marrow’/exp OR marrow) OR intravertebral) AND (‘edema’/exp OR edema) OR vertebral) AND (‘edema’/exp OR edema) OR intravertebral) AND (‘oedema’/exp OR oedema) OR vertebral) AND oedem* OR ‘endplate’/exp OR endplate) AND chang* OR ‘endplate’/exp OR endplate) AND (‘oedema’/exp OR oedema) OR ‘end plate’/exp OR ‘end plate’) AND (‘oedema’/exp OR oedema) OR ‘endplate’/exp OR endplate) AND (‘edema’/exp OR edema) OR ‘end plate’/exp OR ‘end plate’) AND (‘edema’/exp OR edema) OR vertebral) AND (‘marrow’/exp OR marrow) AND (‘lesion’/exp OR lesion)
#13	modic AND change*
#12	#10 OR #11
#11	degenerative AND disc AND (‘disease’/exp OR disease) OR ‘ddd’/exp OR ddd
#10	#8 AND #9
#9	degenerat* OR herniat* OR sequest* OR protru* OR bulg* OR prolap* OR extru*
#8	disc* OR disk* OR discogen*
#7	#5 AND #6
#6	chronic* OR persist* OR continu*
#5	#1 OR #4
#4	#2 AND #3
#3	‘pain’/exp OR pain OR ‘agony’/exp OR agony
#2	((lumbar OR lower) AND (‘back’/exp OR back) OR ‘back’/exp OR back OR low) AND (‘back’/exp OR back)
#1	((low* AND (‘back’/exp OR back) AND (‘pain’/exp OR pain) OR lbp OR ‘back’/exp OR back) AND (‘pain’/exp OR pain) OR ‘lumbago’/exp OR lumbago OR lumbar) AND (‘spine’/exp OR spine) AND (‘pain’/exp OR pain)

**TABLE A3:** CINAHL (EBSCO)

1	Low* back pain OR LBP OR back pain OR lumbago OR lumbar spine pain
2	Lumbar OR lower back OR back OR low back
3	Pain OR agony
4	2 AND 3
5	1 OR 4
6	Chronic* OR persist* OR continu*
7	5 AND 6
8	Disc* OR Disk* OR discogen*
9	Degenerat* OR herniat* OR sequest* OR protru* OR bulg* OR prolap* OR extru*
10	8 AND 9
11	degenerative disc disease OR DDD
12	10 OR 11
13	Modic chang*
14	vertebral body marrow OR vertebral marrow OR intravertebral edema OR vertebral edema OR intravertebral oedema OR vertebral oedem* OR endplate *chang* OR endplate oedema OR end*‐*plate oedema OR endplate edema OR end*‐*plate edema OR endplate NEAR/3 lesion* OR endplate NEAR/3 change* OR vertebral marrow NEAR/3 lesion*
15	13 OR 14
16	12 OR 15
17	MRI OR magnetic resonan*
18	16 AND 17
19	Pyogen* OR infect* OR inflam* OR discitis OR spondylodiscitis OR Cutibacterium *acne** OR C acne* OR bacteri* OR Propionibacterium OR P* ***acnes*** OR anaerobic bacteri* OR low‐virulent
20	Oral antibiotic* OR amoxicillin‐clavulanate OR amoxicillin OR amoxicillin‐clavulanat* OR Bioclavid
21	7 AND 18 AND 20
22	7 AND 18 AND 19 AND 20
23	7 AND 19 AND 20
24	21 OR 22 OR 23

**TABLE A4:** Allied and complementary medicine (OVID)

1	Low* back pain OR LBP OR back pain OR lumbago OR lumbar spine pain
2	Lumbar OR lower back OR back OR low back
3	Pain OR agony
4	2 AND 3
5	1 OR 4
6	Chronic* OR persist* OR continu*
7	5 AND 6
8	Disc* OR Disk* OR discogen*
9	Degenerat* OR herniat* OR sequest* OR protru* OR bulg* OR prolap* OR extru*
10	8 AND 9
11	degenerative disc disease OR DDD
12	10 OR 11
13	Modic chang*
14	vertebral body marrow OR vertebral marrow OR intravertebral edema OR vertebral edema OR intravertebral oedema OR vertebral oedem* OR endplate *chang* OR endplate oedema OR end*‐*plate oedema OR endplate edema OR end*‐*plate edema OR endplate adj3 lesion* OR endplate adj3 change* vertebral marrow adj3 lesion*
15	13 OR 14
16	12 OR 15
17	MRI OR magnetic resonan*
18	16 AND 17
19	Pyogen* OR infect* OR inflam* OR discitis OR spondylodiscitis OR Cutibacterium *acne** OR C acne* OR bacteri* OR Propionibacterium OR P* ***acnes*** OR anaerobic bacteri* OR low‐virulent
20	Oral antibiotic* OR amoxicillin‐clavulanate OR amoxicillin OR amoxicillin‐clavulanat* OR Bioclavid
21	7 AND 18 AND 20
22	7 AND 18 AND 19 AND 20
23	7 AND 19 AND 20
24	21 OR 22 OR 23

**TABLE A5:** Cochrane library

1	Antibiotics
2	Chronic low back pain
